# Interaction of Synthetic Cannabinoid Receptor Agonists with Cannabinoid Receptor I: Insights into Activation Molecular Mechanism

**DOI:** 10.3390/ijms241914874

**Published:** 2023-10-03

**Authors:** Sergei Gavryushov, Anton Bashilov, Konstantin V. Cherashev-Tumanov, Nikolay N. Kuzmich, Tatyana I. Burykina, Boris N. Izotov

**Affiliations:** 1 Engelhardt Institute of Molecular Biology, Russian Academy of Sciences, Vavilova Str. 32, Moscow 119991, Russia; 2 Institute for Translational Medicine and Biotechnology, Sechenov First Moscow State Medical University, 2-4 Bolshaya Pirogovskaya Str., Moscow 119991, Russia; anton_bashilov@mail.ru (A.B.); alknaphel199502@gmail.com (K.V.C.-T.); burykina58@mail.ru (T.I.B.); bn38@mail.ru (B.N.I.); 3 Skolkovo Institute of Science and Technology, Bolshoy Boulevard 30, Bld. 1, Moscow 121205, Russia; 4 The Maurice and Vivienne Wohl Institute for Drug Discovery, Weizmann Institute of Science, Rehovot 7610001, Israel; nnkuzmich@gmail.com

**Keywords:** cannabinoid receptor I, GPCR activation mechanism, apo receptor model, agonist ligand binding, MD simulations of CB1 receptor activation, steered MD

## Abstract

Synthetic cannabinoid receptor agonists (SCRAs) have become a wide group of new psychoactive substances since the 2010s. For the last few years, the X-ray structures of the complexes of cannabinoid receptor I (CB_1_) with SCRAs as well as the complexes of CB_1_ with its antagonist have been published. Based on those data, SCRA–CB_1_ interactions are analyzed in detail, using molecular modeling and molecular dynamics simulations. The molecular mechanism of the conformational transformation of the transmembrane domain of CB_1_ caused by its interaction with SCRA is studied. These conformational changes allosterically modulate the CB_1_–G*_i_* complex, providing activation of the G*_i_* protein. Based on the X-ray-determined structures of the CB_1_–ligand complexes, a stable apo conformation of inactive CB_1_ with a relatively low potential barrier of receptor activation was modeled. For that model, molecular dynamic simulations of SCRA binding to CB_1_ led to the active state of CB_1_, which allowed us to explore the key features of this activation and the molecular mechanism of the receptor’s structural transformation. The simulated CB_1_ activation is in accordance with the previously published experimental data for the activation at protein mutations or structural changes of ligands. The key feature of the suggested activation mechanism is the determination of the stiff core of the CB_1_ transmembrane domain and the statement that the entire conformational transformation of the receptor to the active state is caused by a shift of alpha helix TM7 relative to this core. The shift itself is caused by protein–ligand interactions. It was verified via steered molecular dynamics simulations of the X-ray-determined structures of the inactive receptor, which resulted in the active conformation of CB_1_ irrespective of the placement of agonist ligand in the receptor’s active site.

## 1. Introduction

The cannabinoid type 1 receptor (CB_1_) is a G-protein-coupled receptor (GPCR) in the central and peripheral nervous systems [1]. CB_1_ is one of the most abundant GPCRs in the brain [2], where its activation in the axon terminals of neurons inhibits neurotransmission via presynaptic mechanisms [3]. The agonists of CB_1_ affect cognition, motivation, memory, analgesia, and motor function [4]. The ligands that activate CB_1_ are divided into structurally distinct endogenous cannabinoids (endocannabinoids) and exogenous ligands. Endocannabinoids are lipid-signaling molecules involved in the natural regulation, whereas exogenous ligands are poorly water-soluble and include compounds such as (–)-trans-∆^9^-tetrahydrocannabinol (∆^9^-THC)—the principal psychoactive component of cannabis—and numerous synthetic cannabinoid receptor agonists (SCRAs) intended to elicit psychoactive effects. Despite the diversity of the latter compounds, all of them bear common structural features [5].

A few years ago, the crystal structures of CB_1_ complexes with its agonist [6] and antagonist [7] ligands were obtained using X-ray crystallography. They revealed the binding of exogenous agonist ligands in the extracellular area of the GPCR channel (the orthosteric binding pocket). A structural comparison of CB_1_ complexes with the agonist and antagonist ligands bound clearly demonstrates the active and inactive conformations of CB_1_ indicated by the transverse movement of α-helix transmembrane 6 (TM6) that allosterically modulates the G-protein binding to the GPCR receptor. Further reported crystal structures of CB_1_ complexes with its agonist ligands include the interactions of CB_1_ with the G*_i_* protein [8,9].

In ref. [8] two structures of the complexes of CB_1_ with its agonists were compared to a structure of the CB_1_–antagonist complex. The analysis was supplemented via molecular dynamics (MD) simulations. Finally, some important conclusions about the receptor activation mechanism were drawn. They include the existence of a single active conformation of CB_1_ for the different agonists bound, a crucial role of the stacking interactions of hydrophobic residues of TM2 with the ligand, a deep hydrophobic pocket in the protein structure to bind the ligand aliphatic chain, a possible role of the GPCR “toggle switch” for CB_1_, and a list of the receptor’s residues interacting with the ligand [8]. Such unique features of CB_1_ as the absence of strong atom pair interactions with the agonist ligand and their common hydrophobic nature were also mentioned. However, the authors concluded the incompleteness of the CB_1_ activation description.

In ref. [9], the processes of CB_1_ activation were compared with those for cannabinoid receptor 2 (CB_2_). The study was also supplemented with intense computer MD simulations of the ligand binding. Some new details of the conformational rearrangement at the receptor activation were clarified. In particular, there were important details of the interactions between the residues of the TM5 and TM7 α-helices described, as well as those of TM2 and TM6 during the receptor activation. An important suggestion about the external binding site of endocannabinoids was discussed. However, despite the authors claim that their results reveal the activation mechanism, the entire process of allosteric modulation caused by the ligand binding is not quite clear yet. It especially concerns the role of particular fragments of exogenous agonists in the protein conformational changes at the receptor activation.

Despite the fact that numerous SCRAs that have been in the market for the past decade are structurally heterogeneous, they share common structural features and comprise four subunits: head, linker, core, and tail [5]. The aromatic cores identified are typically indoles or azaindoles, although pyrroles, thiazoles, and napthalenes have also been reported. Linkers connect the core and the head. Typical linkers are ketones, amides, and esters. Heads can be presented by various cyclic and acyclic functional groups. Their size rarely exceeds 10 Å. The tail can be an aliphatic chain sometimes bearing an ω-halogen atom. Such a chain can include a benzene ring. The aliphatic chain length does not exceed seven carbon atoms. More importantly, this chain cannot be shorter than four carbon atoms [5] since the ligand albeit binds but ceases to activate the CB_1_ receptor [10,11,12].

In the present study, we have explored the role of the subunits of the CB_1_ exogenous agonists and the involvement of the ligand’s environment in the ligand–receptor interaction. Based on the published crystal structures of the CB_1_ complexes with its agonist and antagonist ligands and using computer molecular modeling with both the classic and biased MD simulations techniques, we simulated the process of allosteric modulation in CB_1_ for the agonist ligand binding. In other words, we simulated a structural transformation of the receptor from its inactive state to the active-state conformation. Additionally, modeling the ligand modifications and the mutations of protein–amino acids allowed us to verify the role of ligand subunits and clarify the role of particular amino acid residues in the CB_1_ activation process. Finally, this analysis allowed us to determine the stiff core of the transmembrane domain of CB_1_ and suggest a molecular mechanism of the entire conformational transformation of the receptor at its activation caused by the agonist ligand binding. This mechanism was verified via steered molecular dynamics simulations applied to an X-ray-determined structure of the inactive receptor and pointed to mimic the suggested key effect of ligand binding. The resulting active conformation of the receptor appeared irrespective of the placement of agonist ligand in the active site, which proves that this effect is a sufficient condition of CB_1_ activation.

The paper is organized as follows. All steps of the computational study are described in Section 2. In its Section 2.1 we compare the docking of numerous known agonists and antagonists of CB_1_ inside the orthosteric binding pocket of the receptor in its active and inactive states. We conclude that there is a structural region in the receptor where the interactions with the ligands should be similar for both the agonist and antagonist ligands bound. In Section 2.2 we compare the receptor structures known from the X-ray data complexes, superimposing an active-state structure on an inactive-state one with minimal RMSD for this region, which is referred to as the “basement”. It allows us to see the principal structural transformation between the active and inactive conformations of CB_1_. In Section 2.3 we describe a modeled conformation of the inactive receptor in apo form. Within the “basement”, it coincides with the receptor’s conformations in both the active and inactive states but is intermediate between them in some area. In that area, the structural transformation of the receptor from its inactive-state conformation (known from the complexes with the antagonist ligand bound) to the active-state conformation has to overcome a high potential barrier. This modeled structure of CB_1_ is in an inactive-state conformation where it is stable, but the barrier of its transformation into an active state of the receptor at the agonist ligand binding is noticeably lower. The last allows us to simulate the receptor activation at the agonist binding via MD and, using mutations of the amino acid residues and structural changes of the agonist ligand, allows us to draw conclusions about the molecular mechanism of the structural transformation of the receptor (Section 2.4, Section 2.5, Section 2.6 and Section 2.7). Finally, the suggested activation mechanism is explored via steered molecular dynamics in Section 2.8.

## 2. Results

### 2.1. Docking of Different CB_1_ Ligands into the Receptor in Its Active-State and Inactive-State Conformations 

The previously published crystal structures of the active [6] and inactive [7] conformations of CB_1_ allow us to (i) inquire whether the active state is unique for the different and diverse agonists bound and (ii) compare the two conformations to find out the key structural changes. The first question was raised in ref. [8], where it was suggested that the receptor adopted two discrete states: active and inactive. A necessary condition of the active structure’s uniqueness is a possibility to dock any known agonist ligand into the receptor in the active conformation and verify the ligand’s stable binding to the protein molecule (at least for exogenous ligands). As will be shown, an analysis of the docking of antagonist ligands to the CB_1_ molecule can improve our understanding of the conformation changes between the inactive and active states of the receptor.

For the computer docking of ligands to the molecule of CB_1_, nine agonist ligands were selected [5,13,14,15,16,17,18,19,20,21,22,23,24]. A list of them is given in Table 1, along with five inverse agonist (antagonist) ligands [25,26,27,28,29] also selected for docking.

**Figure 1:**
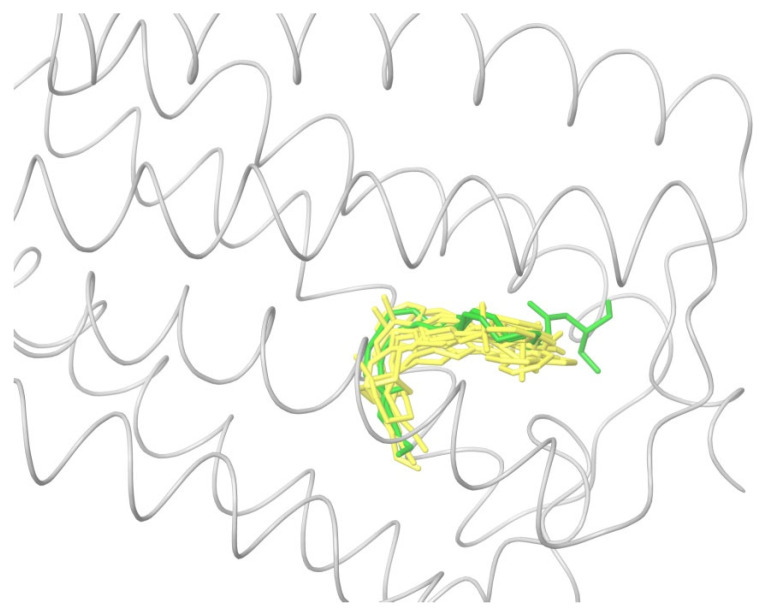
Docking of exogenous agonists from Table 1 into the structure of activated CB_1_ obtained from the crystal complex with AM11542 (yellow). Docked endogenous cannabinoid (anandamide) is shown in green.

**Figure 2:**
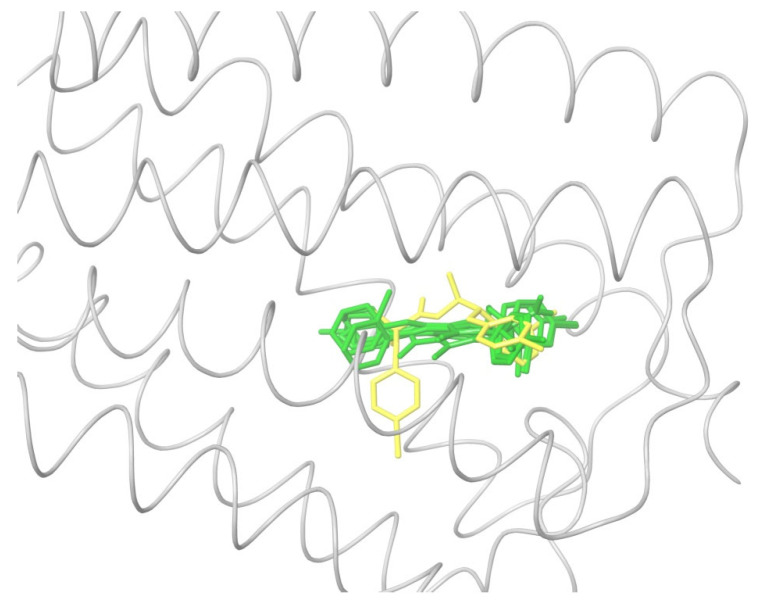
Docking antagonist ligands (green) listed in Table 1 into the structure of activated CB_1_ obtained from the crystal complex with AM11542. Only one ligand could penetrate into the agonists’ tail binding pocket (marked in yellow).

All the ligands from Table 1 were docked into the crystal structure of CB_1_ in its active conformation (from complex with agonist AM11542, PDB ID: 5XRA) [6]. The initial placement of ligands was performed using the Glide XP [30] in the region of the agonist AM11542 binding site. Then, the modeled placement of the ligand was equilibrated via a 50 ns run of the MD simulations of the complex with CB_1_ at the restrains imposed on the atoms of the protein backbone (the Desmond software, release 2017-1) [31]. The final locations of the agonist and antagonist ligands are shown in Figure 1 and Figure 2, respectively.

In the following text, we simplify the description of CB_1_ agonist structures and divide them into the head and tail only. A generalized new “head” includes the commonly adopted “head”, “linker”, and “core” (see colored structure A-834735 in Table 1). As seen in Figure 1, all agonists are docked in the binding site of CB_1_ in the same manner. Their heads interact with hydrophobic residues Phe170, Phe174, Phe177, and His178 of α-helix TM2. This interaction, as an important contribution to the agonist binding, was well established earlier [8]. For brevity, we will further name the aromatic interactions of TM2 with the ligand’s head as the “hydrophobic lock”. As described in ref. [8], in the active conformation of CB_1_, the TM2 helix moves towards the ligand binding pocket, closing the lock. All ligands have their heads’ carbonyl oxygen atoms or hydroxyl groups in the proximity of the side chain of Ser383 to form a non-covalent bond with its hydroxyl group. Another feature of the agonists’ placements shown in Figure 1 is the similar location of their tails. All of them occupy the deep and narrow hydrophobic pocket where the aliphatic chain of agonist AM11542 is located in the crystal structure of the complex [6]. It confirms a noticed important role of this binding pocket [8].

As seen in Figure 2, the attempts to dock antagonists for the active conformation of CB_1_ led to a different picture. One feature clearly distinguishes the placement of antagonists from the docking of agonists: the tail binding pocket is usually empty in the case of antagonist ligands. Since antagonists possess structural subunits similar to the tails of agonists (Table 1), the emptiness of this deep hydrophobic pocket means a high binding energy cost, and such docking, even restrained by the fixation of the protein backbone chain, cannot be regarded as favorable.

**Figure 3:**
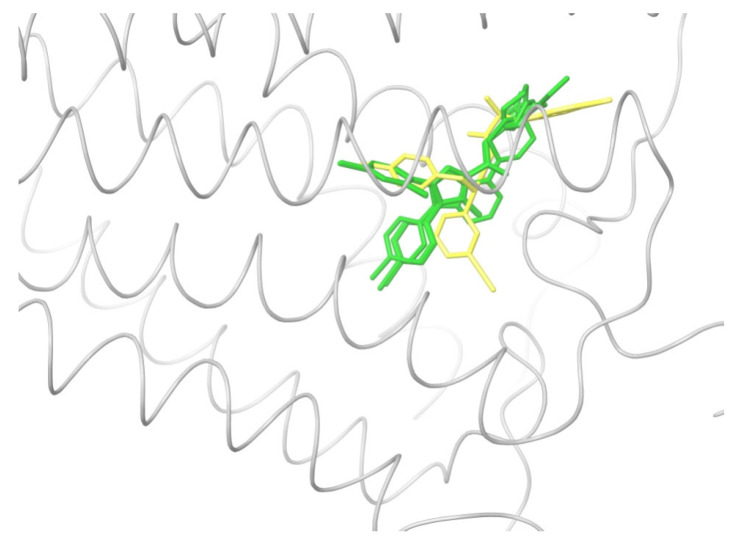
Docking antagonist ligands (green) from Table 1 into the structure of inactivated CB_1_ taken from crystal complex with antagonist. Similar locations of all ligands except for one marked in yellow.

The last conclusion was confirmed when we placed antagonist ligands into inactive CB_1_ taken from a crystal complex of such an antagonist with the receptor (PDB ID: 5TGZ) [7]. The docking procedure and MD equilibration were similar to the ones described above. The results of the antagonists docking into CB_1_ in an inactive state are shown in Figure 3. One can see that the tail-binding hydrophobic pocket is always occupied by the branched subunits of the ligand molecules. Moreover, the placements of all antagonists are quite similar.

A peculiar feature of the occupation of the narrow and deep “tail-binding hydrophobic pocket” observed for both agonist and antagonist ligands gives us a possibility to compare the active and inactive conformations of CB_1_. Since this is the only common feature of agonist and antagonist complexes with CB_1_, one may assume that protein chains surrounding this peculiar binding hydrophobic pocket should be found in similar conformations. Indeed, they interact with and bind similar hydrophobic subunits of different ligands. The tail-binding hydrophobic pocket is surrounded by the extracellular parts of α-helices TM3, TM4, and TM5. Thus, on comparing the conformations of CB_1_ from crystal complexes with agonist and antagonist, it is reasonable to superimpose the protein main chains in the two conformations only for those three α-helices within the hydrophobic pocket region.

### 2.2. Superimposing Active- and Inactive-State Structures of CB_1_

**Figure 4:**
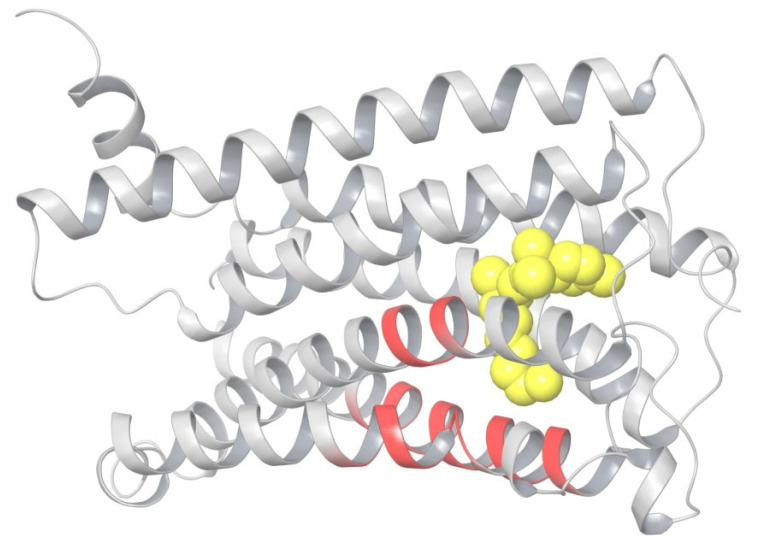
Intramembrane residues of TM3, TM4, and TM5 (red) near the tail binding pocket in the active state conformation of CB_1_. The agonist ligand is shown in yellow.

In this section, we consider such a superposition of the known crystal structures of cannabinoid receptor I that reveals the spatial difference between the active and inactive receptor conformations to a maximum extent. As mentioned before, the expected region where the protein surroundings have to be the least different at the binding of both agonists and antagonists is the envelope of the deep and narrow hydrophobic pocket accommodating the ligand’s tail (Figure 1 and Figure 3 and the blue-marked parts of the chemical structures in Table 1). Considering only the protein residues within the transmembrane region [7,32,33] and excluding the movable TM6 helix, one obtains an approximate picture of the tail-surrounding residues, as shown in Figure 4. Certainly, such a choice is arbitrary to some extent and can be varied plus–minus a couple of residues for each chosen fragment of the protein backbone. It is important that the superimposed chains include fragments of only three α-helices: TM3, TM4, and TM5. We take residues 195–199 (TM3), 243–249, (TM4), and 275–289 (TM5). The last fragment might seem to be chosen too long, as the end of TM5 is shifted at the protein activation [9]. Anyway, the superposition for shorter fragments of TM5 leads to quite similar results. As was mentioned in previous studies [8] and is further discussed in the present work, the very spatial packing of TMs is rigid enough for each conformation of CB_1_ determined via the ligand bound: all seven transmembrane α-helices are glued by numerous mutual molecular contacts to form the central channel. Taking this into account, we can explain the inclusion of a short fragment of TM4 rather for increasing the structurally stable region chosen, as this fragment adjoins to the region of residues of TM3 and TM5 interacting with the ligand’s tail. In addition, the near loop TM4–TM5 is very stiff; it involves an S-S bridge. Further we justify the choice of this structural stability region, considering its peculiar structural features.

**Figure 5:**
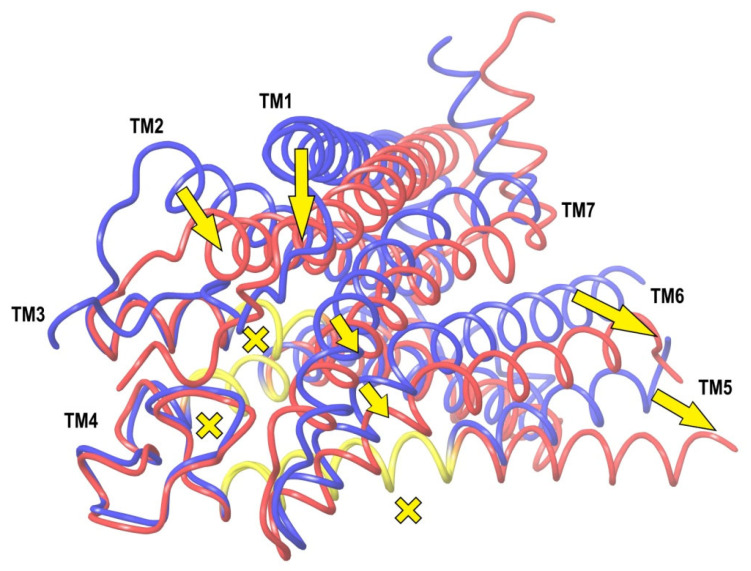
A superposition of crystal structures of CB_1_ in its inactive (blue) and active (red) states. The RMSD of the backbone atoms are minimized in the region marked in yellow. Arrows indicate movements of the transmembrane α-helices, crosses denote coincided ones.

A picture of the superposition of the crystal structure of CB_1_ in its active-state conformation (from PDB entry 5XRA) on its inactive state (from PDB entry 5TGZ) is shown in Figure 5. The superimposed regions to minimize the RMSD of the main chains are marked in yellow, and the observed discrepancies in packing α-helices TM1, TM2, TM6, TM7, and at the end of TM5 are depicted using arrows. Among all seven α-helices, the residues of TM1 and TM4 have no direct interactions with the agonist ligand. 

The structural changes during the activation of CB_1_ are rather evident in Figure 5. Indeed, the “downward” movement of TM1 is due to the removal of the “upper long arm” of the antagonist ligand (see Figure 3). The “downward” movement of TM2 is caused by the attraction of residues F170, F174, F177, and H178 of the TM2′s “hydrophobic lock” to the “generalized” head of the agonist ligand. This interaction was well described earlier [8]. The movement of TM6 provides the allosteric modulation of the G-protein binding. The immobile TM3, TM4, and most parts of TM5 we will further call the “basement”. A key movement causing the allosteric shift of TM6 is a conformational change in the neighboring TM7. In fact, TM6 is just pressed out by the movement of TM7 towards the basement (TM5). At least in part, this movement of TM7 is caused by similar movements of the adjoining TM2 and TM1. TM7 and TM2 (as well as other neighboring α-helices) have numerous molecular contacts of side chains of their residues. All helices form the cylindrical shell of the central transmembrane channel of the receptor and this shell is rigid enough due to the self-consistency of the packing of adjoining α-helices.

How self-consistent the two conformations of CB_1_ are can be verified via MD simulations. We have carried out a simple modeling experiment to check how densely each conformation is packed in terms of the interactions between neighboring α-helices. The replacement of TM6 (residues 332–364), taken in its active-state conformation by TM6 in the inactive-state conformation (in respect to TM5 and TM7) and vice versa, led to the restored original conformation for less than 50 ns MD simulations of CB_1_ immersed into the membrane. Such computer simulations for the replaced TM6 were repeated for the models with ligands bound and when the ligands were removed from the structure of the complex. The stability of the original conformation did not depend on the presence of the ligand. 

Looking at Figure 5, one can raise a simple important question. If one removes the antagonist ligand from the receptor’s channel, some movement of TM1 in the direction shown in Figure 5 should obviously take place. As the ligand’s surroundings in the receptor channel are hydrophobic, the vast vacant space cannot just be filled with the solvent. Then, some movement of TM2, similar to the one shown in Figure 5, and perhaps, of TM7, should also occur. But, it cannot result in the picture corresponding to the active state of CB_1_, otherwise an empty channel would lead to the activated receptor. Thus, the question of which interactions of the agonist ligand with the receptor finalize the protein transformation into its active state remains unresolved. In the following, we describe a study dedicated to this quest.

### 2.3. Model of a Sensitive-to-Agonist-Binding Conformation of Unliganded CB_1_

When ligands are removed from complexes with the seven-transmembrane (7TM) helices domain of CB_1_, the receptor structures present themselves as coherent, self-consistent, and metastable, irrespective of the receptor’s active-state or inactive-state conformations (Figure 5). It can easily be demonstrated via MD simulations. If one removes the antagonist ligand from the inactive structure (PDB ID: 5TGZ) and starts MD simulations of the transmembrane domain of the remaining empty receptor (immersed into a membrane) during hundreds of nanoseconds of an MD run, the protein tertiary structure remains mainly intact except for the denser packing of TM1 due to the ligand removal. If one docks the agonist ligand into this structure, no transformation into the active-state conformation known from the crystallographic data will occur for any reasonable time of the simulation run (more than a thousand nanoseconds). Equally, if one just removes the agonist ligand from the active-state structure of CB_1_, even a very long run of MD simulations of such an empty receptor in the membrane (up to a thousand nanoseconds) indicates no visible structure transformation, leading to the disappearance of the active-state conformation of TM6. This means that high potential barriers have to be overcome at forming the conformation of CB_1_ caused by the protein–ligand interactions. (Such barriers should be distinguished from the free energy barrier at the transfer of a ligand molecule from the bulk solution into the receptor’s orthosteric binding pocket—it is also high enough [9]). It is clear that the barrier of the receptor’s structural transformation is caused by the numerous mutual interactions of the adjoining residues of neighboring α-helices of the densely packed channel protein.

If we built a stable model of the 7TM domain of CB_1_ in an inactive apo from which can be transformed into the receptor’s active conformation at the computer simulations of the agonist ligand binding, it would be possible to explore the nature of the allosteric modulation mechanism via ligand modifications or mutations of the receptor residues. At least it could clarify the roles of various ligand–protein interacting groups. It is important to emphasize that this might not be the natural apo form of the receptor, but in respect of allosteric modulation, this stable structure should correspond to an inactive conformation of CB_1_. Then, with some probability, this particular inactive conformation appears in reality, and one could analyze the features of receptor activation due to agonist binding at least for this conformation. Further, in this subsection, it is described as modeling a stable inactive structure of apo receptor that is somehow between the active and inactive conformations of CB_1_ known from the X-ray-determined complexes. Admitting that there might be other inactive apo forms of the receptor with lower free energy and looking more different from both its known active and inactive structures, we try to start from searching for a stable apo form of CB_1_ which is somehow on the way of the transformation of the receptor from its inactive conformation to the active one.

As mentioned above, a clear cause of the structural transformation of CB_1_ from its inactive state to the active one is the interactions of TM2 with a head of the agonist ligand (“hydrophobic lock”)—one of the main sites of ligand binding [8]. It leads to the displacement of TM2 (Figure 5). To some extent, we can also add interactions between the agonist ligand and TM6 or TM7. The displacements of TM1 and the intracellular parts of TM5 and TM6 are far beyond the direct interactions with the agonist ligand; therefore, we regard them as secondary ones. But, those very outward movements of TM6 and TM5 are of importance since they provide the allosteric modulation at the G-protein binding. One can assume that for building a model of the receptor in apo form that is sensitive enough to be easily transformed into the final active conformation by the agonists bound, it is necessary to diminish the high potential barrier at the shift of TM2 (Figure 5). The barrier itself is caused by dense TM2–TM3 and TM2–TM1 interactions along the α-helices at the corresponding movement of TM1.

The desired model of the 7TM domain of apo CB_1_ should include the following features. TM6 and TM7 still remain in the inactive-state conformation, but the geometry of TM1 and TM2 is noticeably closer to their final active conformation at the binding of the agonist ligand. Such a model can be built in several ways. For example, after removing the antagonist ligand from the inactive-state crystal complex, one can apply a metadynamics simulation procedure to force the shift of TM2 and TM1 towards their desired locations in the active conformation of CB_1_. Then, the void space of the hydrophobic channel in its extracellular region will be noticeably diminished. This metadynamics simulation procedure is described in Section 4 and the results are shown in Supplementary Figure S1. In our study, a different procedure has also been applied and we finally modeled a structure of the apo receptor in a bit more complicated way. It involved simple molecular modeling and a series of computer MD simulations to equilibrate the receptor without a ligand in the membrane environment. The details of modeling are described in Supplementary Section S2. An obtained structure of the unliganded receptor was applied in a further study described below. It is essential that this apo form of the transmembrane domain of CB_1_ is (i) in an inactive conformation and (ii) metastable (retains its conformation for more than 1000 ns of MD simulations in the membrane environment).

**Figure 6:**
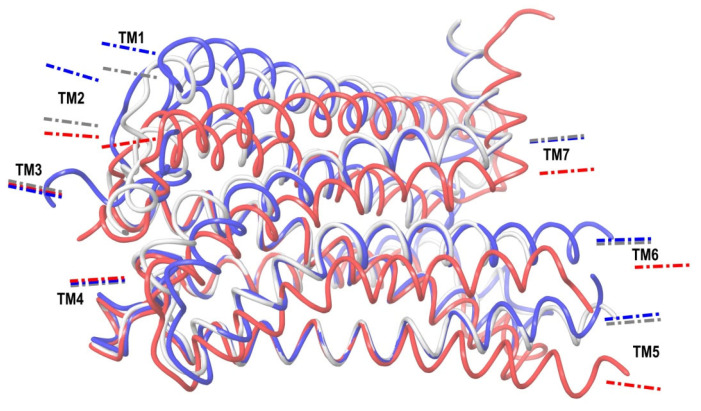
Comparison of CB_1_ structures from the crystal structure of complex with antagonist (blue, inactive-state conformation), from the crystal structure of complex with agonist (red, active-state conformation), and modeled apo-receptor structure (white). The structures are superimposed on each other as in Figure 5. Directions of axes of the α-helices are depicted as short dashed–dotted lines.

A superposition of the active-state, inactive-state, and modeled apo conformations of CB_1_ are shown in Figure 6. As seen, α-helices TM3, TM4, TM5, TM6, and TM7 in the apo-state conformation almost coincide with those in the inactive-state conformation. On the contrary, TM2 is quite close to (but not coincides with) TM2 in the active-state conformation. Helix TM1 is found somewhere between its conformations in the active and inactive states.

The molecule of the CB_1_ agonist AM11542 from the reported X-ray structure of the complex [6] was docked into the model of apo CB_1_ (the coordinate file of the resulting complex was published earlier [34]). As seen from a structure comparison shown in Figure 6, such a complex appears to be structurally not much different from the crystal complex from the X-ray data. The ligand placement does not undergo serious steric collisions. 

**Figure 7:**
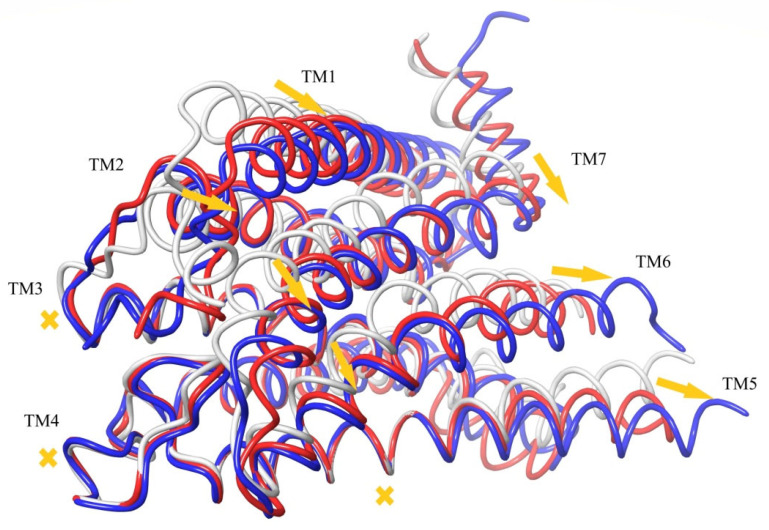
Results of MD simulations of CB_1_ in modeled apo-receptor conformation with docked agonist AM11542. The apo-receptor model is shown in grey, and the final conformation after a 400 ns MD run in red. The structure of CB_1_ in the active state from crystal complex with AM11542 [6] is shown in blue. The structures are superimposed at minimization of RMSD of protein backbone atoms of the “basement” (residues 195–199, 243–249, and 275–289). The α-helix movements are depicted using arrows. Immovable α-helices of the “basement” are marked using crosses.

The results of 400 ns MD simulations of the new complex of CB_1_ in the apo-receptor conformation with AM11542 are shown in Figure 7. One can see that all the receptor structure transformations into the active state (Figure 5) have occurred after this MD run. Actually, the crucial ones such as the movement of TM7 pressing out the allosteric α-helix TM6 take about 200 ns of simulation time. We should not be confused by the incomplete outward movement of TM6 at its breakage point in the intracellular area, as a part of the chain (307–338) is removed from the model. As a result of this model distortion, a rather hydrophobic intracellular entrance into the channel appears to be exposed to the solvent. (A similar bent of TM6 near the breakage of the chain (residues preceding Leu345) can be observed for the prolonged MD simulations of the crystal complex of CB_1_ with AM11542). Another thing of crucial importance: the exact coincidence of the final location of TM7 with its geometry in the crystal complex and the long coincidence of TM6 (residues 345–367) with TM6 from the active-state X-ray structure.

Among other important structural changes, we should mention the move of TM1 towards its placement in the crystal structure and a displacement of TM2 due to the accommodation of its “hydrophobic lock” interacting with the head of the agonist ligand. In Figure 7, all movements of α-helices are shown in respect to the immovable “basement” of TM3, TM4, and TM5 marked in Figure 4 and Figure 5. The residues of this “basement” interact with the tail of the ligand.

The “basement” fragments of the receptor’s backbone of TM3, TM4, and TM5 chosen for the structures’ comparison consist of a significant part of the stiff core of the TM7 domain. It is clearly seen from the calculations of the root mean square fluctuations (RMSF) over the MD trajectories. The hydrophobic core of globular proteins and the structural core of channel receptors should possess properties of relatively stiff structure with the mean thermal motion noticeably lower than for the backbone of other residues. The last is clearly seen from an RMSF analysis for the model of the receptor in apo form (Supplementary Figure S3). The “basement” chosen as the surroundings of the hydrophobic tails of the agonist and antagonist ligands bound is a significant part of the stable fraction of the backbone observed in the MD simulations. A similar RMSF distribution for the chain of alpha carbon atoms of CB_1_ is obtained at the simulations of the X-ray-determined structures of CB_1_ complexes with agonist and antagonist ligands (PDB entries 5XRA and 5TGZ). 

One feature of the initial ligand docking into this apo form of the receptor was useful for further research: there was only a tiny movement of the ligand molecule relative to the “basement” after a 400 ns MD run (less than 1 Å). The backbone chain of the “basement” itself was immobile as well (RMSD less than 0.5 Å). The resulting structure is not changed if the heavy atoms of the backbone chain of the “basement” are spatially anchored via a restraining potential. The MD simulations were repeated when the heavy atoms of both the ligand and backbone of the “basement” were fixed by the restraining potential. It led to a receptor conformation that was close to the structure of the ligand–receptor complex obtained from the simulations with the free ligand. It is not so if the ligand is structurally modified (see below). 

The obtained transformation of the 7TM domain of CB_1_ into its active-state conformation due to the agonist ligand binding allows us to explore the entire mechanism of the allosteric modulation. It can be studied in a series of particular MD simulations with this modeled structure of CB_1_ when the receptor and ligand are deliberately modified.

### 2.4. The CB_1_ Activation Molecular Mechanism

The activation of CB_1_ at the agonist ligand binding described in the previous section occurs within a short simulation time (~200 ns) due to the fact that in the inactive-state metastable apo conformation, the potential barrier of closing the hydrophobic lock is essentially diminished in comparison with, e.g., the conformation of CB_1_ taken from the crystal complex with the antagonist ligand. It is important to note that this entire transformation is reversible. Indeed, when we removed the agonist ligand from the final active structure shown in Figure 7 (in red color) and started the MD simulations of the empty receptor, the original apo-receptor structure was restored in a 200 ns MD run. This directly proves that in these simulations, the activation is entirely driven by the protein–ligand interactions.

It should be noted that we could not obtain the inactive-state conformation of the unliganded receptor directly via the MD simulations of its active conformation from the crystal complex [6] when the agonist ligand is removed. The simulation experiments showed that additional potential barriers were in the intracellular region of the model distortion, where the protein chain is broken for α-helices TM5, TM6, and TM7. This part of the X-ray data structure of the active-state receptor was replaced by the active-state structure obtained in the simulations of our apo-receptor model with the agonist ligand (residues 195–414). After that, the inactive apo-receptor structure reappeared in 200 ns MD simulations of the empty receptor in the membrane despite some structural differences in TM1 and a slight difference in the backbone chain of TM2 (red and blue structures in Figure 7).

The second computational experiment confirmed the crucial role of the “hydrophobic lock”. We started the MD simulation run for the apo-receptor conformation after docking AM11542 into its binding pocket, but at that time, the protein backbone atoms of the TM3–TM5 “basement” (residues 195–199, 243–249, and 275–289) and, on the other hand, the entire α-helix TM2, were restrained to move due to an imposed restraining potential. (Further in the text, we call such a restriction as the “fixation” of a protein backbone chain fragment or another atomic group). Thus, we anchored TM2 in respect to the “basement”, preventing the shift of TM2 as shown in Figure 7. As a result of a long MD simulation run, no active conformation was observed. In fact, the structure of the complex remained intact. Therefore, the shift of TM2 is of crucial importance for the receptor activation. The TM2 displacement (from 1.5 Å for Ala160 to 3.8 Å for Val171) is smaller than the movement of adjoining TM7 (3.5–5.0 Å for residues 377–398) and TM1 (up to 7.7 Å for Ser114). This shift is the final accommodation of the “hydrophobic lock” (F170, F174, F177, and H178) relative to the “basement”. Thus, an obstacle for this final accommodation totally blocks the receptor’s structural rearrangement.

The role of the “hydrophobic lock” accommodation in respect to the geometry of the “basement” can easily be proven by several other simulation experiments. In the first one, we remove the entire tail of the ligand and only its “head” remains. But now, the “head” is spatially fixed at the MD run, as well as the receptor’s “basement”. As mentioned above, after the simulations with complete AM11542, the ligand is almost immovable relative to the “basement”. The results of 200 ns MD simulations of the apo-receptor model with the ligand’s head at such restraints are close to those shown in Figure 7, where the active-state conformation appears. On the contrary, if we release the head of AM11542 and the ligand has no “tail”, no active conformation of CB_1_ is obtained. Instead, the “head” interacts with the “hydrophobic lock” of TM2 and notably moves from its initial placement. And finally, if we fix the “tail” of the ligand in respect to the fixed “basement” and remove the ligand’s “head”, there is no active conformation of the receptor after the MD simulations either.

**Figure 8:**
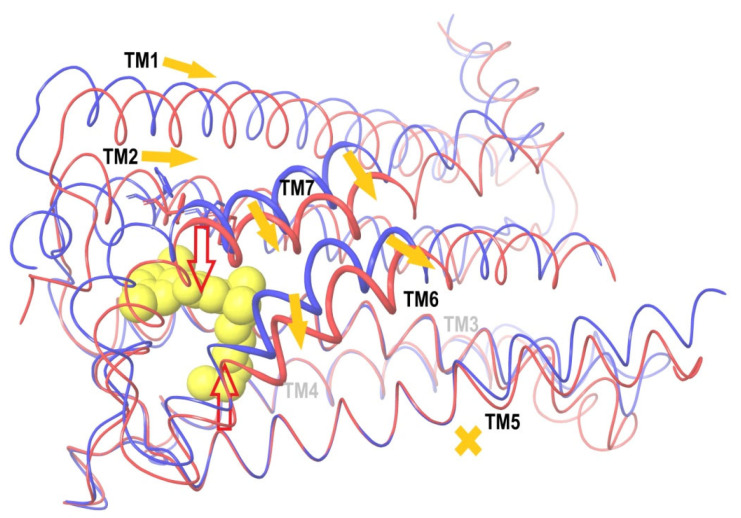
An explanation of the activation mechanism. The AM11542 agonist ligand molecule is shown in yellow. Red arrows denote hydrophobic interactions of the ligand “tail” with TM3 and TM5 and the ligand “head” with the “hydrophobic lock” of TM2 (its residues F170 and F174 are shown). The backbone for the apo-receptor model conformation is marked in blue, and results of MD simulations of this model with the ligand are shown in red. The depicted thick part of the backbone of TM6 and TM7 marks residues chosen for RMSD evaluation to detect the receptor activation. Yellow arrows show shifts of TM1, TM2, TM6 and TM7 relative to the “basement” of TM3, TM4 and TM5 (yellow cross). See the text for further details.

Thus, the entire molecular mechanism of CB_1_ interaction with its endogenous ligands looks very simple. The ligand “head” attraction to the “hydrophobic lock” of TM2 together with the ligand’s tail binding to the “basement” (α-helices TM3, TM4, and TM5) force α-helices TM1 and TM7 to move towards the “basement”, mainly due to a shift of TM2 relative to this “basement”. This contraction of the “cylindrical channel shell” of TMs pushes out α-helix TM6, providing the allosteric modulation of the G-protein binding. This scheme is shown in Figure 8. The shift of the backbone of TM2 is towards TM7 and is almost tangential to the “basement”. The movement of the side chains of TM2′s “hydrophobic lock” (especially Phe170 and Phe174 in the case of ligand AM11542) pushes the neighboring residues of TM7 towards the “basement” along the direction of the ligand’s tail aliphatic chain. The direct interactions of TM7 residues with the ligand head (such as a hydrogen bond of Ser383) also take a part in this shift of TM7 towards the basement.

**Figure 9:**
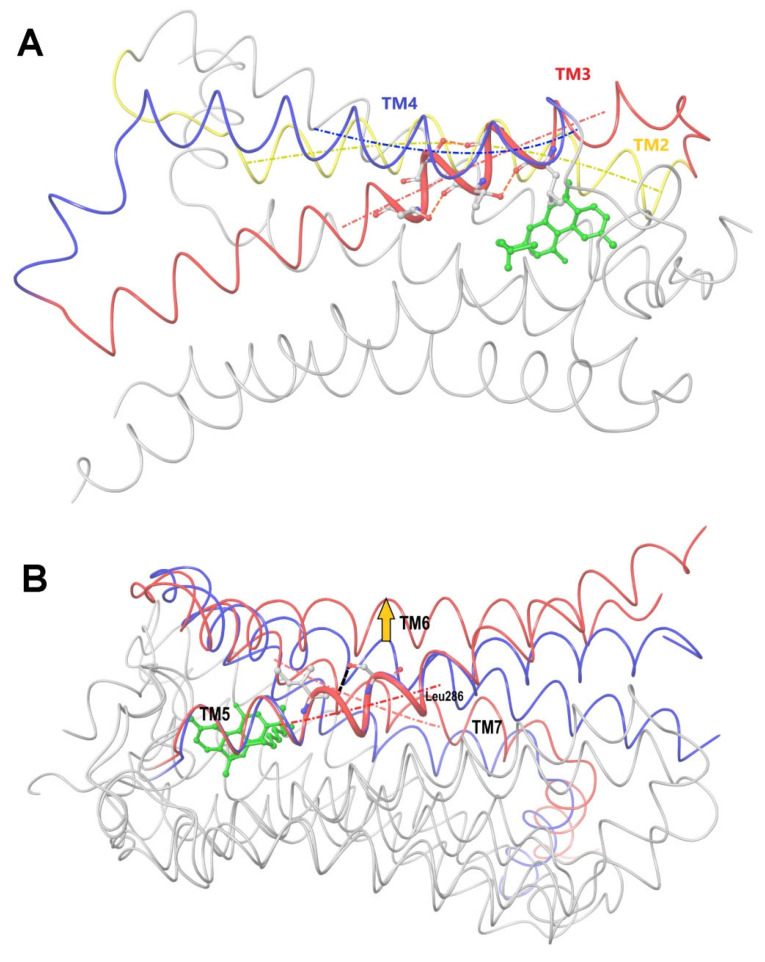
(**A**) Approximate projection of the mean force acting on the TM3–TM4 part of the receptor’s hydrophobic core (TM3–TM5) due to TM2–ligand head interactions along the aliphatic chain of the ligand tail (looking towards us) and perpendicular to the planar ligand head. Alpha helix TM3 (red) is sandwiched between TM2 (yellow) and TM4 (blue). Its part reinforced by three additional hydrogen bonds is marked thick. Axes of the α-helices are depicted using dashed–dotted curves. (**B**) Approximate projection of the mean force acting on the TM5 part of the receptor’s stiff core due to movement of TM7 towards TM5. The projection is along the aliphatic chain of the ligand tail (looking towards us) and perpendicular to the planar ligand head. Superposition of the inactive-state (blue) on active-state (red) conformations of CB_1_ taken from crystal complexes of CB_1_ with antagonist and agonist ligands. The structures are superimposed at “basement” residues 195–199 (TM3), 243–249 (TM4), and 275–289 (TM5). Axes of helices TM7 (in the active state) and TM5 are shown as dashed–dotted lines. The agonist ligand AM11542 is colored in green. The “reinforcing” hydrogen bond between the side chain of Ser284 and Ile280 is shown as a dashed line. The backbone of the loaded part of TM5 is marked thick. Leu286 in CB_1_ corresponds to the proline residue in other A-GPCR receptors. This residue separates TM5 into the rigid and flexible parts. Due to ligand–protein interactions, the mean force acts on TM7 towards fixed TM5 along the line of the observer’s sight, pressing TM6 out (yellow arrow).

At this point, we should carefully examine the previously determined “basement” which interacts with the alkyl chain of the ligand tail. Indeed, the three α-helices TM3, TM4, and TM5 form a very stiff and densely packed structure. The hydrophobic core of their mutual interactions includes the following residues: 197–212 (TM3) contacting with 275–289 (TM5); 190–201 (TM3) contacting with 244–251 (TM4); and 247–251 (TM4) contacting with 275–279 (TM5). In fact, the TM3–TM4 hydrophobic interactions involve the entire length of each α-helix. In addition, TM4 is connected to TM5 via hydrogen bond Tyr275–Ile247. The extracellular loop TM4–TM5 is fixed via S-S bridge Cys257–Cys264. The dense packing of TM3 and TM4 is maintained via additional interaction Asp213–Tyr224 at the intracellular loop connecting them. In the suggested activation mechanism, there is a preferential direction along the alkyl chain of the tail of ligand AM11542 perpendicular to the approximate plane of its head’s cycles. Along this line of sight, the projections of axes of α-helices TM4, TM3, and TM2 in the active state conformation cross at approximately one point, giving the “loaded nodes”: residues 244–251 (TM4) against 194–200 (TM3) against 166–173 (TM2) (Figure 9A). We assume “loaded” as a tension is expected (red arrows in Figure 8) approximately along this line. An indirect evidence of this loaded node of TM3 (which is between TM2 and TM4 in this projection) is the fact that the backbone chain of α-helix TM3 is reinforced exactly in this region via three additional hydrogen bonds: Thr197–Leu193, Ser199–Gly195, and Thr201–Thr197. In addition, the TM4 conformation is strengthened via Pro251. Looking at the residues of TM5 neighboring to the two “loaded nodes” of TM3 (194–200) and TM4 (244–251), it is seen that they involve residues 275–283. And we again find the reinforcing of the backbone via an additional hydrogen bond: Ile280-Ser284. Thus, according to the crystal structure of CB_1_ in its active-state conformation, we see a “reinforced” basement of heavily packed and interacting helices, where the conformation of residues 194–200, 244–251, and 275–283 is expected to be very stable. Comparing to our initial choice for the structures’ superposition (195–199, 243–249, and 275–289), one can see that they coincide except for the longer length of the stable part of TM5.

It should be noted that the kink point of TM5 in CB_1_ is Leu286. For other class A GPCR proteins, there is a proline residue at this position and it is a kink point of TM5 [8]. It is important to note that this residue is at the crossing of the TM5 and TM6 axes’ projections in the inactive state along the line of tension—in the active state, TM6 is pressed out and moves outwards. It not only justifies our initial choice of the superposition residue intervals, but also casts some light on how to detect the active state in the result of the simulations. As mentioned above, the very beginning of TM6 is subjected to interactions due to the breakage of the protein backbone in the intracellular domain of the simulated model, and we even truncated TM6 to Ile339 to avoid a computationally consuming breakage of the “ionic lock” [9] between the moving TM6 and TM3 (Asp338–Arg214). But, the active state of CB_1_ should be indicated via the movement of TM6 and TM7 in respect to TM5, rather in the extracellular and membrane regions than at the very beginning of TM6 where the simulation model is not correct due to the breakage of the protein backbone. Thus, looking at Figure 7, it is natural to calculate the RMSD of the TM6 and TM7 displacement from the inactive state of our apo-receptor model to the active state, counting the residues of TM6 and TM7 lying above TM5 before its kink point of Leu286. “Above” means the direction of the tension approximately along the alkyl chain of the ligand tail (Figure 9B). It gives an approximate interval of residues Val351–Thr391. The “helical part” of this interval is marked in Figure 8 and it was used for the RMSD calculations for comparison with the active- and inactive-state crystal structures of the receptor. The results of the MD simulations for different ligand modifications or mutations are collected in Table 2, where the active state is detected via RMSD in this interval of residues when the alignment of Cα atoms of the compared structures includes “basement” residues 195–199, 243–249, and 275–289.

One important consequence from the suggested explanation of the activation mechanism is that the geometry of the agonist tail link to the ligand head’s core cannot be too flexible, as it is expected to be highly strained at the binding (red arrows in Figure 8). Indeed, in the cores of numerous synthetic cannabinoid receptor agonists of all classes [5], there are rigid unsaturated structures such as indoles and azaindoles which do not leave the tail’s chain connection with such cores showing too much flexibility. Structural rigidity of, e.g., cannabinomimetic MDMB-fubinaca in its characteristic L-shape configuration could result in its high efficacy [8]. One can also conclude that the flexible endocannabinoids could not serve as such a tensed connection between TM2 and the “basement” of TM3 and TM5, even if they were water soluble. Such lipid-soluble endocanabinoids should interact with CB_1_ in quite a different manner (see Section 3).

In the following subsections, the receptor–agonist ligand interactions will be considered in detail and the role of some important protein residues will be described.

### 2.5. Free Energy Costs of the Ligand Tail and Head Bindings

The model of apo receptor described above allows us to understand the role of ligand functional groups or particular protein residues in the receptor activation. If a change in the ligand or a mutation of a residue affects the transformation of the receptor into its active state, one may suspect their important role in the activation of the receptor.

In the activation mechanism discussed, the role of the ligand’s “tail” binding is crucial. Indeed, it is bound to the receptor in a narrow and deep hydrophobic pocket formed by the side chains of Leu193, Thr197, Phe268, Ile271, Tyr275, Leu276, Trp279, and Met363 [8]. All of these residues, except for Met363, belong to the structural “basement” (TM3 and TM5 or the stiff loop between TM4 and TM5 reinforced via an S-S bridge). It is well known that neither the “tail” aliphatic chain nor its cyclic analogs (the fluorobenzyl one, for instance) cannot be too short, or otherwise a substance ceases to be cannabinomimetic [5]. If the chain is shorter than the propyl group, the binding of the ligand notably decreases [23].

To verify this dependence, we used ligand AM11542 from the crystal structure of the agonist–CB_1_ complex. There were several 200 ns scale MD simulations of the model of apo receptor immersed into the membrane performed, including the docked molecule of AM11542 with its tail shortened. All simulations with tail’s aliphatic chain shorter than five carbon atoms failed to result in an active conformation of the receptor (Table 2). For the five carbon atoms and longer chains of the tail, the final simulated structure was quite similar to the results of the simulations with AM11542 intact (Figure 6 and Figure 7).

The dependence of the binding free energy of AM11542 on the length of its aliphatic tail was studied via the MD simulation technique using the original crystal structure [6] of the CB_1_–AM11542 complex. The details of those ∆∆G calculations are given in Section 4. The values of the ∆∆G binding given in Table 3 suggest that the hydrophobic (i.e., mainly of entropic origin) free energies of the agonist tail binding are high enough. The free energy cost of the entire tail binding exceeds 14 kcal/mol. When the tail is truncated to the five carbon atom chain, the binding free energy loses more than 5 kcal/mol, but when it is truncated to four carbon atoms, the energy is reduced by half. One can conclude that a loss of the tail binding energy exceeding 7 kcal/mol might lead to the impossibility to provide enough of a tightening force, as shown in Figure 8, to shift TM1, TM2, and TM7 in their active conformation placements and, as a result, to push TM6 out.

The change in the binding free energy at the thermodynamic transformation of the entire ligand AM11542 into its tail without the ligand’s head gives us the energy of the head binding to the “hydrophobic lock”. Such was performed for the crystal structure of the CB_1_ complex with AM11542 (Section 4, “Alchemical free energy calculations”). The binding energies appeared to be even higher than those in the case of the tail interactions. A disappearance of the entire planar structure of the head subunit of AM11542 (Table 1) gives us the head’s ∆∆G binding energy as exceeding 17 kcal/mol. From this value, the two outer cyclic structures of the head (the third one is linked to the tail) take about 10 kcal/mol (the ∆∆G difference between the whole and truncated heads). Thus, one can see that, according to the crystallographic structure of the complex with the agonist ligand, the ligand’s head is “glued” to TM2 even better than the tail to its hydrophobic pocket. In both cases, the energy of the hydrophobic interactions is high (from 14 to 17 kcal/mol).

The simulated binding energies of the tail and head are close to each other (14 and 17 kcal/mol, respectively)—in fact, they differ almost within the accuracy of the ∆∆G simulations. It indicates the equality of mean forces acting on the ligand’s head and tail in opposite directions. Moreover, the binding energy is spent to the transformation of the receptor to its active-state conformation, not to the tension of the bonds at the contacts of the atomic groups of the protein and ligand. (The difference of the binding free energies of the head and tail will be spent on such local tension.) It directly follows from our previously mentioned experiment when we fixed the ligand head via a restraining potential and the ligand had no tail. The basement was anchored relative to the ligand head position as well. MD simulations for such ligand fixation were performed for our apo-receptor conformation. When we fixed the position of the ligand’s head, which was taken after the docking calculations, an incomplete transformation of the receptor into its active-state structure was observed. But, when we took the corrected location of the fixed ligand’s head that followed from the MD simulations of the model with the free whole ligand (ligand position difference is less than 1 Å), the transformation of the receptor into its active-state conformation via the interactions with only such a fixed ligand’s head was undistinguished from the results of the simulations with the whole ligand. (In Table 2: the “model with fixed AM11542 without its tail” and the “model with precisely fixed AM11542 without its tail”.) Now, the energy of the tail interactions was accepted by the restraining potential (no visible shift) that provided the “tail” force marked in red in Figure 8, whereas the “head” force remained. No special interactions of protein residues with the ligand’s tail in addition to its retaining in the binding pocket was needed to transform the protein into its active site—the tail was removed and only the forces acting on the head fixed in respect to the basement in its final binding position were enough to do this. And even a slight incorrectness of the fixed head position led to a loss of the head binding energy due to the local deformation of the contacting atomic groups, not to the global structural rearrangement of the receptor.

### 2.6. Energy of “Toggle Twin Switch”

Alpha helix TM6 that allosterically modulates the binding of the G protein to CB_1_ contains Trp356—the so called “toggle switch”. This residue is conservative for GPCR receptors. Previous studies applying molecular simulations have confirmed its important role in the activation of e.g., rhodopsin [35,36], β_2_-adrenergic receptor [37,38], M2 muscarinic acetylcholine receptor [39], or opioid receptor [40,41]. In refs [8,9], the role of Trp356 is discussed. Especially, it concerns its stacking interactions with Phe200 in the reported crystal structure of CB_1_, where the receptor is in its inactive state with the antagonist ligand bound. The latter “twin toggle switch” interaction was assumed to play an important role in the interaction with the antagonist ligands as well as in the fixation of the inactive receptor structure. That conclusion is justified by the fact that the mutations of Phe200 affect the binding activity of CB_1_ [42].

Our apo-receptor model is sensitive enough to be transformed into an active conformation at the agonist ligand binding. It allows us to explore the role this “twin toggle switch” plays in the receptor activation. As follows from the MD simulations, when agonist ligand AM11542 was bound to the apo receptor with mutated Trp356 or Phe200 to glycine, no transformation into the active state of the receptor occurred and the receptor conformation remained intact (Table 2). But, on the other hand, the statistical analysis of trajectories suggests that the conformation of residue F200 is rather unstable in this conserved inactive conformation of CB_1_ for the model studied. The latter facts rather suggest that preventing or impeding the receptor’s structural transformation by these mutations cannot be explained via the direct stacking interactions between Trp 356 and Phe 200, although such interactions take place in the inactive structure of CB_1_.

To resolve this question, there were MD simulations performed of the free energy perturbation to mutate both Trp356 and Phe200 into alanine residue. The results of the free energy changes ∆G are collected in Table 4. The crystal structure of inactive CB_1_ was used (PDB ID: 5TGZ) with the ligand removed.

As seen in Table 4, the replacement of the side chain of Ala356 via Trp356 decreases the free energy by 6.94 kcal/mol in the absence of the Phe200 side chain (Ala200) and by 6.97 kcal/mol in its presence. Thus, the appearance of the side chain of Phe200 gives only −0.03 kcal/mol. The difference in the appearance of the side chain of Phe200 caused by the presence of the Trp356 side chain is −3.12 − (−2.45) = −0.67 kcal/mol. The difference between the two ∆∆G values obtained (−0.03 and −0.67) reflects the accuracy of the simulations as these values should be equal to each other. Anyway, we can see that the free energy of the stacking interactions between the side chains of Phe200 and Trp356 in the protein environment is low: it is less than 0.7 kcal/mol. Thus, the main contribution to a decrease in the free energy at the appearance of the toggle switch Trp356 side chain is due to the entropic cost of filling the cavity (~7 kCal/mol), not due to the weak stacking interactions with Phe200 (less than 1 kcal/mol).

The negligible role of Phe200 in the inactive-state conformation also follows from the MD simulations. As described above, after agonist ligand docking, the MD simulations of CB_1_ in the apo-receptor conformation resulted in the active-state conformation of the receptor. Then, we removed the ligand molecule and repeated the “inverse” simulations, but Phe200 was mutated to Gly200. The initial inactive-state conformation of the “empty receptor” was restored in a 200 ns MD run.

Anyway, the mutations of the “toggle twin switch” residues can prevent or impede the activation of the receptor at the agonist ligand binding [42]. At least, it is confirmed in our experiments with the apo-receptor model including the docked AM11542 (Table 2). When mutated to glycine, Gly356 or Gly200 does not allow the receptor’s transformation into the active conformation for 200 ns run, whereas it takes place when Trp356 or Phe200 remain intact. But, it suggests the significant increase of the free energy of the entire system due to expanding the void space in the absence of the residue’s side chain. It would be a purely entropic contribution, not the energy of the stacking interactions between Trp356 and Phe200. Indeed, the entropic cost due to the absence of the side chains of the two residues is high enough at 7 and 3 kcal/mol (Table 4). When this emptiness expands, one could expect an increase in these values that might be notably higher than ~ 0.7 kcal/mol of the 356–200 stacking interaction energy.

In a similar manner, an entropic contribution due to the neighboring locations of Trp356 and the ligand’s tail is observed. Indeed, as follows from Table 2, when the ligand is spatially fixed by the restraining potential, a partial activation of the receptor takes place when Trp356 is mutated to glycine. On comparing the two structures of the complexes averaged over the last 50 ns of the MD simulations (Trp356 is mutated to glycine, the ligand is free to move versus the ligand is fixed), it is seen that in the case of the free ligand, the ligand is shifted towards the “hydrophobic lock” of TM2. To some extent, it looks like the picture when the free-ligand tail is cut off, i.e., now the TM2– ligand interaction energy is spent on a deformation of the hydrophobic lock residues, not to the transformation of the entire 7TM domain because of the weaker ligand tail binding (see Figure 8). Thus, Trp356 “interacts” with the neighboring ligand, but rather in terms of the entropic contribution of filling voids into the free energy cost.

The interaction of Phe200 with the tail of the agonist ligand is negligible. We do not mean the free energy of the interaction of the side chain of Phe200 with the hydrophobic pocket of the tail—it might be noticeable enough. The difference of the Phe200 interaction energies is considered when the void pocket is replaced by the pocket containing the atoms of the ligand’s tail. First, it follows from our experiment with the tail removal together with the ligand head fixation that there is no difference of the MD simulations’ results for the receptor–ligand complex with the free whole ligand and with only its fixed head (see above). Second, it follows from the direct ∆∆G simulations at the mutation of Phe200 into Ala200. In addition to the calculations reported in Table 4, the thermodynamic transformations of the residue were performed in the presence of the ligand and in its absence (at Trp356 intact). The difference was within the statistics error (less than 0.5 kcal/mol). The role of Phe200 seems to be different: it could prevent the penetration of ligands into the channel, providing their binding inside the orthosteric binding pocket.

### 2.7. Role of Phe379 and Ser383

As mentioned above, the hydrophobic pocket of the ligand tail binding includes residues Trp279, Tyr275, Leu276, Ile271, Phe268, Met363, Leu193, and Thr197. The “hydrophobic lock” of TM2 interacting with the head of the antagonist ligand includes residues Phe170, Phe174, Phe177, and His178. From the outer part of the channel, the ligand’s head can also interact with residues Leu193, Phe268, Phe379, Leu359, Ser383, and Phe189. All of them were mentioned in the earlier studies [8,9]. Here, we should emphasize an important role of residue Phe379 on the receptor activation, which has not been described before.

**Figure 10:**
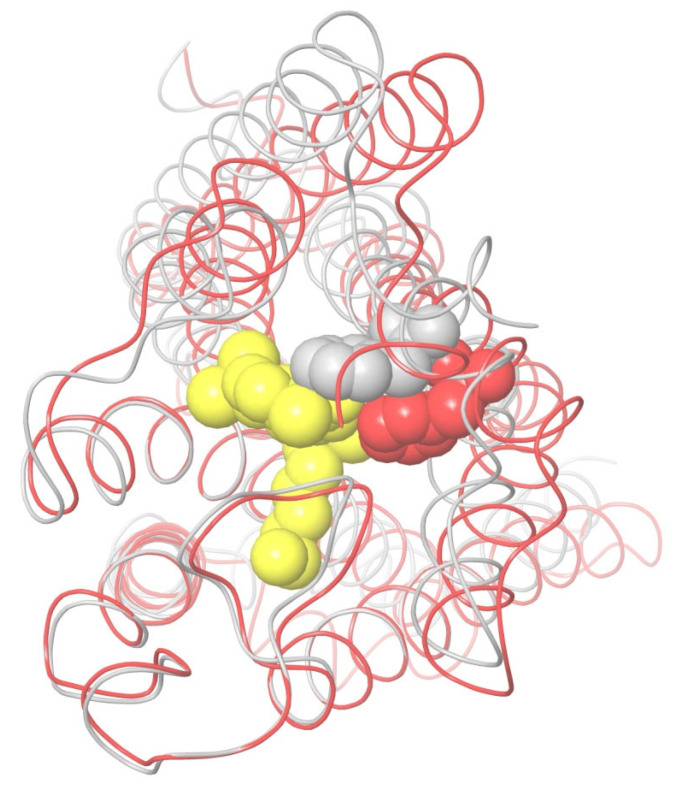
Interaction of Phe379 with the head of ligand. The residue is shown in gray for the apo-receptor model and in red in the active conformation of the receptor interacting with agonist ligand (yellow).

As shown in Figure 10, this residue not merely interacts with the ligand head, but it also exhibits noticeable changes its location in the final active structure of CB_1_. As follows from the MD simulations, a mutation of this residue to one with a smaller side chain prevents the transformation of the receptor into its active-state conformation (Table 2). The simplest explanation is a stronger attraction of TM7 due to the free-energy entropic contribution of the released empty space. As a result, TM7 cannot easily move towards the “basement”, as shown in Figure 7. Thus, this interaction of the side chain of Phe379 with the ligand head facilitates the movement of TM7 towards TM5, increasing the pressing of TM7 on TM6.

Ser383 forms a hydrogen bond with the ligand’s head. This bond is present for all agonist ligands (Table 1, Figure 1) as their polar oxygen atoms or hydroxyl groups are always found in close proximity of the serine residue hydroxyl group after the ligand docking calculations. It could indicate the crucial role of this bond in the activation mechanism. This role is revealed via two MD simulations with the apo-receptor model of CB_1_ when Ser383 is mutated to glycine. At the first simulation, AM11542 was fixed via a restraining potential. In the second case, the docked agonist ligand is completely free (the “basement” was fixed via the restraining potential in both cases). The results of the RMSD comparison with the active and inactive conformations of CB_1_ are presented in Table 2. One can see that in the case of the fixed ligand, the hydrogen bond energy itself is not crucial—results are undistinguished from the simulations with the fixed ligand and the “basement” for the apo-receptor model without any mutations. The final receptor conformation is close to its active-state conformation known from the X-ray data. When the ligand was free to move, the mutation of Ser383 prevented the transformation of the receptor into its active-state structure. Moreover, the head of the ligand moved close to the “hydrophobic lock” of TM2 residues Phe170, Phe174, and Phe177. There was a notably shorter movement of the side chains of these residues towards the basement (TM3–TM5); instead, the head of the ligand was distorted and moved towards TM2. (The ligand’s tail was found at the same position as it was from the simulations without the mutation.) In other words, the free energy of the ligand’s head binding to the “hydrophobic lock” was spent not on the structural transformation of CB_1_ due to the move of TM2 and TM7, but on the local deformation of the ligand at the contact of its head and the “hydrophobic lock” of TM2. Therefore, the role of Ser383 is a stiff connection of the ligand’s head to TM7 to transmit the ligand’s interaction energy to the CB_1_ conformation rearrangement via the movement of the side chains of “the hydrophobic lock” residues (Figure 8) and not just to the ligand’s head deformation. This was clearly indicated in the mutation experiments: Ser383 played a crucial role in mediating the ligand specific interactions for the agonists at CB_1_ [43].

Two residues of the moving α-helix TM6 directly interact with the agonist ligand: Leu359 and Met363. A mutation of both of them to glycine did not affect the capability of AM11542 to transform the inactive-state conformation of our apo-receptor model to an active state of the receptor on ligand binding (Table 2). Thus, these interactions should not be regarded important for receptor activation.

### 2.8. Verification of the Suggested Mechanism of CB_1_ Activation by Means of Steered Molecular Dynamics Simulation

An impact of the move of TM7 relative to the stiff core of the trans-membrane domain of CB_1_ on the domain’s conformation was analyzed via the steered molecular dynamics (SMD) [44,45,46]. According to the conclusions drawn above (Figure 8 and Figure 9), the main cause of pushing TM6 to its location in the active conformation of CB_1_ is the displacement of TM7 towards TM5, as shown in Figure 8. The move of the far beginning of TM6 needed for the interaction with the G protein inside the cell is of secondary nature and caused by a shift of the part of TM6 marked as thick wire in Figure 8 in the ligand binding site region. All direct interactions of TM6 with the ligand (including the highly conservative “toggle switch” Trp356) are of secondary importance. In other words, the move of TM7 towards TM5 (to be exact, towards its part corresponding to the stiff core of the receptor domain) has to be a sufficient condition of the receptor activation.

To verify this conclusion, a modification of the SMD method has been used. The structures of CB_1_ in active and inactive conformations were taken from published X-ray data. After equilibrating the MD simulations (100 ns), they were superimposed on each other, minimizing the RMSD for the described above stiff core (“basement”) of the transmembrane receptor domain (residues 195–199 of alpha helix TM3, 243–249 of TM4 and 275–289 of TM5). After this coordinates’ superposition, the SMD simulations were applied to a model of CB_1_ in the membrane environment. The details of the SMD implementation are described in Section 4 (“Steered molecular dynamics”). An artificial external force was applied to the alpha carbon atoms of a chosen part of the TM7 alpha helix (residues 374–392, see above) for the inactive conformation of CB_1_ to move it towards their locations in the active conformation under the condition of retaining the core Cα atoms of the receptor (“basement” described above, residues 195–199 of TM3, 243–249 of TM4, and 275–286 of TM5). Instead of the SMD ensemble averaging over multiple transformations [44], only one move was simulated but slowly enough to keep the system in thermodynamic equilibrium. Therefore, the external force work is the change in the free energy ∆*G* of the system at such a transformation. The last quantity was counted but, in fact, is of no importance, as the study was focused on the comparison of the resulting protein domain conformation with its true active conformation known from the X-ray data.

If the CB_1_ activation mechanism could be reduced to the effects of pressing TM7 towards TM5 (Figure 8), such a move of TM7 relative to TM5 caused by an external force should result in the active CB_1_ conformation independently on the agonist ligand presence in the active site. On the contrary, if the receptor activation is sensitive to the interactions of the side chains of the TM6 residues with the ligand’s atoms, the receptor’s conformational change at this displacement will depend on the presence of an agonist molecule in the receptor active site.

**Figure 11:**
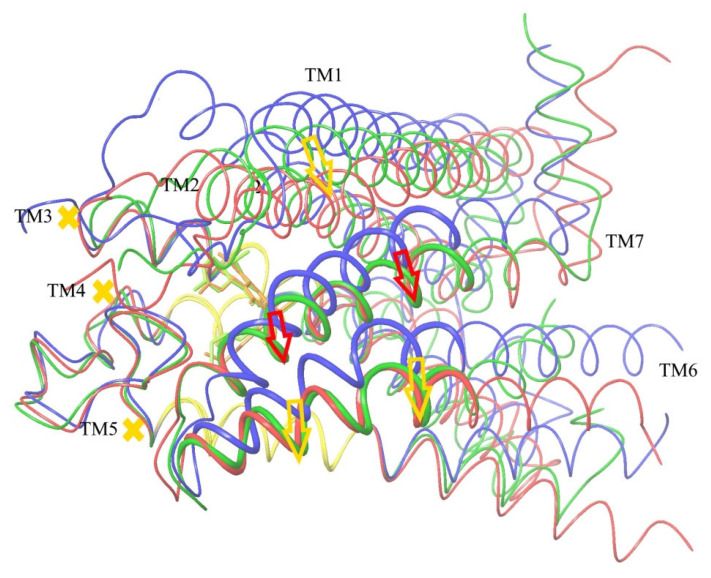
Results of SMD transformation of CB_1_ if there is agonist ligand in the active site. Inactive conformation is shown in blue, true active conformation in red (agonist ligand is shown by thin orange lines), and results after TM7 displacement to their positions in the active conformation in green. Thick wires mark residues of moved residues of TM7 and residues of TM6 chosen for activation detecting. The stiff core of receptor is colored yellow. Red arrows mark the pressing external force. Yellow arrows show resulting shifts of helices.

**Figure 12:**
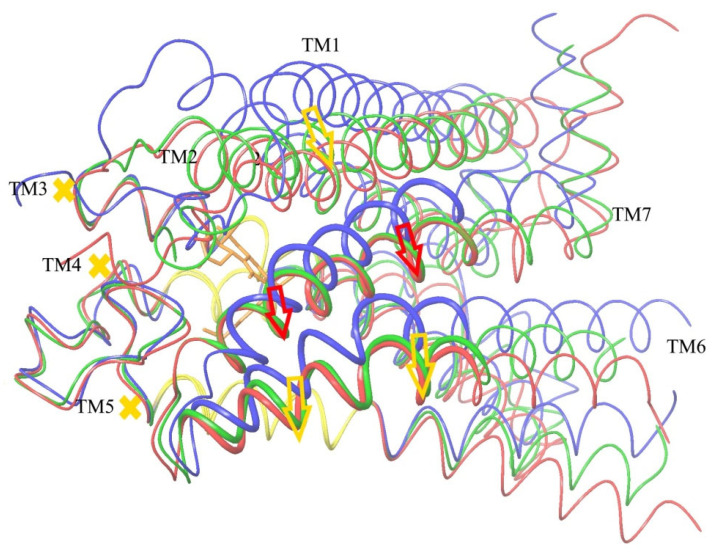
Results of SMD transformation of CB_1_ without agonist ligand. Inactive conformation is shown in blue, true active conformation in red (agonist ligand is shown in thin orange lines), and results after TM7 displacement to their positions in the active conformation in green. Thick wires mark residues of moved residues of TM7 and residues of TM6 chosen for activation detecting. The stiff core of receptor is colored yellow. Red arrows mark the pressing external force. Yellow arrows show resulting shifts of helices.

The results of two SMD simulations to push the alpha carbon atoms of alpha helix TM7 (residues 374–392) to their positions in the active CB_1_ conformation are shown in Figure 11 and Figure 12. In the first case, the agonist ligand was present in the active site (Figure 11), whereas in the second case, it was absent. As seen from the two pictures, the part of TM6 where the activation can be indicated (residues 351–366, see Figure 8) appeared in the same “active” conformation regardless of the ligand’s presence. Moreover, the resulting displacement of alpha helix TM2 strongly interacting with all the agonist ligands (the “hydrophobic lock” interactions, see above) appeared almost the same and correspond to its active-conformation position. After the superposition of the results of SMD in the presence of the agonist ligand (Figure 11) and the structure of CB_1_ in the active conformation, the mean RMSD atoms of the main chain consisted of 0.76 Å for residues 351–366 of TM6, 0.19 Å for residues 374–392 of TM7, and 1.14 Å for residues 170–178 of TM2 (“hydrophobic lock”). In the absence of the ligand (Figure 12), those RMSD were 0.90 Å, 0.17 Å, and 0.88 Å, respectively. In both cases, the RMSD for the retained core of the receptor (residues 195–199, 243–249, and 275–286) was about 0.4 Å. The RMSD averaging was performed for the final step of the SMD transformation over the last 8 ns of simulation time (8000 frames). Thus, the force acting on alpha helix TM7 towards its position in the active conformation on the condition of retaining the protein core is a sufficient condition to transforming the entire structure of the membrane domain of CB_1_ into its active conformation. All deviations observed can clearly be attributed to the incomplete model of the CB_1_ macromolecule (the broken chains of TM6, TM5, and TM8 inside the cell and the broken chain of TM1 outside the cell).

## 3. Discussion

The structural model of the unliganded 7TM domain of receptor CB_1_ allowed us to perform a transformation of the receptor to its active-state conformation via computer MD simulations of the exogenous agonist binding to CB_1_. This model describes just a metastable intermediate state between the known atomic structures of the complexes of CB_1_ with the antagonist and agonist ligands solved from the crystallographic X-ray data. The sensitivity of the model to ligand binding and a possibility of the theoretical modeling of the receptor’s active state have helped us to verify, confirm, and complete the previously claimed features of the receptor activation mechanism [8,9].

They can be summarized as follows:

*(i)*  Any (meta)stable conformation of the seven transmembrane α-helices forms a shell around the solvent-accessible central channel. This quasi-cylindrical shell is quite resistant to the changes of mutual locations of the adjoining helices and looks like a cylindrical shell made of very viscous and, at the same time, elastic substance.
*(ii)*  The active-state structure of CB_1_ is unique for the exogenous agonist ligands bound. It appears at the maximal pressing on α-helix TM6 between TM7 and TM5. This pressing is caused by a shift of TM7 towards the solid core of the transmembrane domain of the receptor. This stiff core is formed by transmembrane helices TM3, TM4, and TM5. The displacement of TM7 relative to the stiff core is due to the interaction of TM2 and TM7 with the ligand bound in the orthosteric binding pocket and anchored to this core. It leads to the shift of the α-helix TM7 towards α-helix TM5, the opposite neighbor of TM6, which takes place along the entire length of TM7 and TM6. It finally pushes TM6 out, forming the conformation of the activated receptor.
*(iii)*  The binding of different inhibitors to the receptor does not lead to a single receptor’s state—they can presumably affect the receptor conformation in different ways to prevent the appearance of the allosterically modulating conformation of TM6. The binding of known antagonists in the channel stretches the cylindrical shell, leaving no possibility for α-helix TM6 to protrude outside. On the contrary, the binding of the exogenous agonists of CB_1_ leads to the macromolecule’s internal tension shrinking the cylindrical shell.
*(iv)*  There are three peculiar features of the agonist ligand structure (in addition to its size and flexibility to the entry at the orthosteric binding pocket). The first one is the high-energy stacking interactions of the ligand head with the “hydrophobic lock” of TM2 (residues F170, F174, F177, and H178). The second feature is the high energy of the hydrophobic interaction of the ligand’s tail with the residues of TM3 and TM5 in a deep and narrow hydrophobic pocket. The third one is a stiff connection between the ligand’s tail and head: this link cannot be flexible. As a result, the connection between the ligand’s core and tail has to be a rigid molecular structure. The latter is observed in reality for all known cannabinomimetics. And, the tail has to be maximally long to strengthen its binding in the hydrophobic pocket. This conclusion agrees with the properties of real cannabinomimetics as well. In fact, the core-tail link of the ligand plays the role of a bowstring between TM2 and TM7 and, on the other hand, TM3 and TM5. The attraction of the TM2–TM7 and TM3–TM5 residues to the ligand molecule shrinks the channel shell along this bowstring, shifting TM1 and TM7 towards TM5 and finally pushing TM6 outward.
*(v)*  The “toggle twin switch” Trp356–Phe200 interaction itself plays no role in retaining the inactive-state conformation of the receptor, although both residues are important for reaching its active state.

Some of these conclusions have been drawn in previously published researches [8,9], but others require comments. One conclusion concerns the “stiff core” of the seven-transmembrane region of the receptor. According to our structural research, this core contains extracellular-side fragments of TM3, TM4, and TM5. They are involved in the surroundings of the agonist ligand orthosteric binding pocket. We called this rigid structure the “basement”, as it interacts with the CB_1_ ligand’s tails. The core includes TM3 and TM4 (except, perhaps, the intracellular loop connecting them), and TM5 from its beginning to residue Leu286, after which TM5 gains flexibility at the structural activation change. The TM4–TM5 loop fixed via an S-S bond could also be added to this “basement”. The nature of this core is revealed via the comparisons of the crystal complexes of CB_1_ with the agonist and, on the other hand, with the antagonist ligands bound. It is also clearly indicated in the thermal motion analysis via the calculations of the backbone atoms’ RMSD for the MD runs of the receptor (Supplementary Figure S3). This hydrophobic region looks mostly stable at the thermal motion—a usual definition of the hydrophobic core for proteins. The kink point or hinge of TM5 (Leu286) corresponds to the proline in other class A GPCR proteins [47]. This structural rigidity means a load in terms of the mean force. Indeed, instead of proline, in CB_1_ we find a strengthening of the rigid part of TM5 near that point via a hydrogen bond of the side chain of Ser284 with carboxyl oxygen of the Ile280 (Figure 9B). The TM4 α-helix is also reinforced via Pro251 in the stiff core considered. The involvement of TM4 in the domain’s structural core explains its role, as TM4 does not directly interact with ligands. It forms the rigid “basement” of the molecular structure via numerous interactions with the highly reinforced TM3. Finally, we draw the conclusion about a different method of superimposing active and inactive conformations of the receptor and a different indicator of its active state (Figure 8, Table 2). The use of TM6 movement as an indicator of the active state in the crystal complexes of GPCRs is unreliable. Coupling with a transducer such as the G protein seems to be necessary for GPCRs to adopt the fully active state [48]. At crystallization, the transducers are replaced by G-protein mimetics, e.g., camelid antibody fragments that bind directly to the intracellular side of GPCR [49,50]; therefore, the conformation of TM6 can be affected by this binding.

Summary (*i*)–(*v*) is also applicable to the highly relative type-2 cannabinoid receptor (CB_2_). Both CB_1_ and CB_2_ are the biological targets of Δ^9^-THC (Table 1) and their seven-transmembrane region sequences have a 68% similarity [51]. The structures of the agonists and antagonists for the two receptors bear features similar to those shown in Table 1 (the “head” and the “tail”). Some compounds are non-selective, although others exhibit selectivity [52]. The X-ray-determined structure of a complex of CB_2_ with agonist AM12033 [9] as well as the cryo-EM-determined structure of a CB_2_-WIN55,212-2 complex [53] reported recently reveals quite similar agonist bindings. The interactions of the “hydrophobic lock” with the ligand head, the anchoring of the ligand’s tail in the deep hydrophobic pocket, the positions of “toggle switch” Trp258, and the neighboring Phe117 of CB_2_ look like the picture described for CB_1_ above.

The ideas about the activation of GPCRs were considered in recent reviews [49,50,54,55]. Despite quite different sequences and the chemistry of the agonist binding, the spatial backbone patterns of the 7TM domains for class A GPCR (such as rhodopsin, β-adrenergic receptors, or μ-opioid receptor) can be superimposed on each other at low RMSD for both active and inactive conformations. It suggests a very similar mechanism of the entire structure transformation that is not completely understood yet [56]. One of the described experimental results was a conclusion about “basal equilibrium” and multiple conformations of the unliganded receptor [57]. The described switching between the inactive states within hundreds of microseconds and a high energy barrier to achieve the active state qualitatively agree with our conclusions from the computer simulations of CB_1_. As described above, the free energy difference at the rearrangement of the receptor topology is about 10 kcal/mol (according to the ligand’s tail and head binding energies). Here, we assume the lower limit of the shortest tails’ binding energy, the equality of the mean forces acting on the head and tail, and the total spending of this energy on the protein structural rearrangement, not on the local tension of the interacting atomic groups. But, the energy barrier at the receptor activation can be much higher than this final difference—this is the cause of the impossibility to reach the active state in the simulations by just replacing the inverse agonist with the agonist ligand in the inactive structure. This is why we built the intermediate unliganded model of the still inactive-state conformation where this barrier was significantly decreased.

The limiting case of the active state observed for the crystal complex of CB_1_ with agonist AM11542 (and achieved in our MD simulations for the apo-receptor conformation with AM11542 docked) does not contradict the fact that other ligands can cause other rearrangements of the 7TM domain structure when we deal with the interactions of GPCR with different G proteins or β-arrestin [55]. It is merely stated that there is a definite structure rearrangement in a case of the full agonist (such as AM11542) corresponding to a GPCR-G_i_ complex. This activation is detected not by the outward movement of TM6 (which depends on the presence of a nanobody or G protein) [58,59], but by the value of the movement of TM7 towards TM5 (more than 3 Å) in a region shown in Figure 8, when we compare the active and inactive structures by means of superimposing the “stiff cores” of the structures. Then, a corresponding movement of the pressed out TM6 should also be about 3 Å within this region. It can be illustrated for other class A GPCRs. In Supplementary Figures S4–S6, such a superposition is performed for the comparison of active and inactive X-ray structures of the β_2_-adrenergic receptor [60,61], the μ-opioid receptor, [62,63], and rhodopsin [64,65], respectively. In all three cases, one can see a picture quite similar to that shown in Figure 8: TM7 and TM6 are moved by about 3 Å towards TM5 in the region chosen for the detection of the activation, i.e., in the fragments of TM6 and TM7 “above” TM5 taken up to the proline residue of TM5, where projections of the TM7 and TM5 axes along the direction of the TM7 shift cross each other (Figure 9B). In all three cases, TM3 is reinforced by two or three additional hydrogen bonds in a region similar to that shown in Figure 9A.

In previous studies [56,66], a similarity of conformation changes at the class A GPCR activation is emphasized, although the changes are described differently. Despite the fact that the difference between that picture and our scheme is a matter of a choice of the “coordinate system” (i.e., what moves relative to what), the description of the “stiff TM core” as an immovable part allows us to see the meaning of the receptor’s structure transformation more clearly. In other works [67], the suggested mechanism of the receptor activation is based entirely on the “molecular switches” buried in the receptor structure (such as the ionic lock between TM3 and TM6 fixing the inactive state). The role of these switches is undeniable, but they only support the main cause of the receptor transformation: the pressing out TM6 by the pushing TM7 towards TM5 of the “stiff core” (Figure 8). The proof of a supplementary function of “molecular switches” was the MD simulations of the activation/inactivation of CB_1_ at the insertion/removal of the agonist ligand to/from the orthosteric binding site, when this ionic lock was just removed (Table 2). In fact, the mechanism seems to be the same for all class A GPCRs, but the energy supply for the movement of TM7 towards TM5 differs according to the different bindings of different ligands. Another indirect suggestion of the pressing-TM6-out mechanism could be an experimentally observed shift to the active conformation of a GPCR under high pressure [68].

The conclusion (*v*) seems to contradict the experimentally established importance of Trp356 for the receptor activation [42]. But, Table 4 suggests not only a weak energy of the Trp356–Phe200 stacking interaction (less than 1 kcal/mol), but also a high energy of the Trp356 mutation to alanine (about 7 kcal/mol). Thus, the change in free energy at the movement of TM6 can also differ noticeably in the presence of the side chain of Trp356 and in its absence. In other words, we should look not at the energy of the Trp–Phe aromatic lock (the “twin toggle switch” energy), but at the difference in the change in the free energy of the entire system at the TM6 outward movement when Trp356 is replaced by Ala356.

In statements (*i*)–(*v*) summarized above, we see a simple molecular mechanism that has no crucial pair interactions (except, perhaps, with Ser383) and has only diffuse “hydrophobic gluing” of the ligand’s head and tail to protein. It rather suggests that we deal not with the evolution of the protein structure pointed to bind exogenous SCRAs, but with something that occasionally fits for some external compounds like ∆^9^-THC. Indeed, according to the discussed mechanism of receptor activation, the molecules of the endogenous cannabinoids (which are apparently a result of evolution) cannot activate CB_1_ from inside the GPCR channel due to their flexibility. One can admit that such a structural feature as the deep pocket of the tail binding inside the channel might also appear occasionally. But, it could hardly have happened since its emptiness had cost a significant increase in the protein free energy. Rather, this hydrophobic pocket could serve as some natural inhibitor binding.

## 4. Materials and Methods

### 4.1. Docking of Ligands into Receptor and MD Simulations

The Protein Preparation Wizard of the Schrodinger suite of programs (Schrodinger INC, New York, NY, USA) was employed to prepare the protein X-ray structures by adding hydrogen atoms and missing side chains and by assigning an ionization state for the amino acid residues and ligands at the physiological pH value. The docking calculations were performed using the Glide XP module of the Schrodinger 2017-1 suite of programs [30]. The receptor grid was generated using a default algorithm and a 10 Å box centered on the receptor’s co-crystallized ligand. All the ligands were prepared using LigPrep [69]. Some of the final placements of ligands were completed manually using molecular modeling software. The membrane region around the receptor was taken from the Uniprot database [32]. The following MD simulations were performed using the Desmond module of the Schrodinger 2017-1 suite of programs [31,70] with a OPLS3 force field [71]. For the periodic boundary condition (PBC) simulations, the transmembrane domain of CB_1_ was placed into a rectangular box with a buffer size of 25 Å. The POPE (310 K) membrane model was applied. The protein charges were neutralized by adding 0.15 M of NaCl with an excess of chloride ions into the TIP3P water. The equilibration of the system was achieved via consequent simulations of Brownian dynamics (10 K) and consequent molecular dynamics (NVT, 10 K; NPT, 10 K; and NPT, 310 K). The final productive MD runs were performed at 310 K in the NPT ensemble. When necessary (as described in the Section 2), the restraining potential (force constant 80 kcal/Å^2^/mol) was applied to the nitrogen and carbon atoms of the protein backbone as well as to the heavy atoms of the ligands.

### 4.2. Alchemical Free Energy Calculations

The ddG calculations were performed using the FEP+ module (Desmond 5.8) of the Schrodinger 2019-2 suite of programs [72,73,74,75]. The biologically irrelevant fragments were removed from the 5XRA and 5TGZ structures, which were prepared using the Protein Preparation Wizard [76,77,78]. The termini were capped, bond orders were assigned, hydrogens were added, and missing amino acid side chains were filled with Prime [76,77,78,79]. The titratable residue protonation states were visually inspected and the complex underwent minimization (RMSD = 0.5 A) using the OPLS3e forcefield [80], which was also used by all subsequent simulations. Since the ligand nitrate group in the 5TGZ complex has not been structurally resolved, the standard precision (SP) Glide docking [81] with core constraint was used for the placement of the complete ligand. All the ligands were prepared using LigPrep [82] and the missing torsion parameters were fit with the ForceField Builder. For the PBC simulation, the complexes were neutralized with Cl- ions embedded in the POPC lipid pre-aligned membrane according to the Orientations of Proteins in Membranes (OPM) database [83] and placed into a SPC water box with buffer sizes of 20, 20, and 15 Å. The system was minimized via 500 ps of Brownian dynamics, keeping the protein and ligand heavy atoms restrained (10 kcal/Å^2^/mol) and then relaxed using the default membrane relaxation protocol. The end temperature was 310 K. The ligands before FEP+ calculation were aligned to the native one (via the maximum common substructure) and perturbation maps with optimal topology have been constructed. Every perturbation consisted of 12 lambda windows that were each 20 ns long in the μVT ensemble for both the ligand and amino acid transformations. Replica exchange with solute tempering (REST), an enhanced sampling technique introduced by Wang et al. [84], was employed. By default, the REST region atoms were the mutated ones. Also, the grand canonical Monte Carlo (GCMC) [85] method was used to enhance the sampling of water molecules in the binding site. Analysis of selected trajectory frames was performed using the reanalyze_fep.py script when necessary. For every edge of the perturbation map, the ddG values were computed using the Multistate Bennett Acceptance Ratio (MBAR) method and were given both with and without cycle closure correction (CCC).

### 4.3. Metadynamic Simulations

They were also performed using Desmond 5.8 of the Schrodinger 2019-2 suite. The ligand was removed from the PDB 5TGZ complex and the system was built and relaxed as described above. The further staged relaxation was carried out at 310 K in the NPT ensemble and included a 5 ns simulation with the protein heavy atoms restrained (10 kcal/Å^2^/mol) and a 1 ns simulation keeping the protein backbone atoms restrained (5 kcal/Å^2^/mol), followed by a 10 ns simulation of unrestrained protein. We used the Nosé–Hoover thermostat with a relaxation time of 1.0 ps and the Martyna–Tobias–Klein barostat with a relaxation time of 2.0 ps for temperature and pressure control, respectively. The relaxed CB_1_ inactive apo conformation was aligned to the CB_1_ molecule in the active state (5XRA), having amino acid residues of 195–199, 243–249, and 275–289 as the anchor points. We chose two collective variables (CV) describing the transformation: the distance between the C-alpha atoms of F381 and V179 (CV1), and the angle between the C-alpha atoms of the T125, P113, and F102 residues (CV2). The decrease in CV1 characterized the approaching of TM3 to its position in the CB_1_′s active-state conformation, while the increase in CV2 elevated the resolved part of the N-end loop, removing it from the path of the V179 move and facilitating the helix shift. The CV1 values changed to 13 Å, which approximately corresponds to the CV1 value of the 5XRA structure, and the CV2 values increased from 55.2 degrees. The simulations themselves were run at 310 K in the NPT ensemble for 100 ns with the same thermostat and barostat as stated above. The height of the Gaussian repulsive potential was set to a 0.03 kcal/mol, the time interval for the addition of the Gaussian repulsive potential was 0.09 ps, and the RMS width of the Gaussian repulsive potential was 0.05 Å for CV1 and 2.5 degrees for CV2. The frames were recorded every 10 ps. One of the frames corresponding to the PES minimum was checked for stability via a 500 ns conventional MD run at 310 K in the NPT ensemble.

### 4.4. Steered Molecular Dynamics Simulations

A simplified technique of the steered molecular dynamics [44] (SMD) was implemented, applying a designed Python script editing the data of the input files of the Desmond software (Desmond 5.8 of the Schrodinger 2019-2 suite) [72,73]. The main goal of the SMD simulations was the “infinitely” slow move of a subset of restrained atoms along the given trajectories. In other words, the centers of the retaining external potentials applied to these atoms are very slowly transferred from the starting positions of the moved atoms to their destination positions. The SMD implementation chosen included positioning the center **r**_0_ of a harmonic restraining potential *U*^ext^ = *k*(**r** − **r**_0_)^2^/2, which is applied to a moved atom so as each new location of **r**_0_ appeared on the line connecting the averaged previous position of the atom <**r**>^(*i*)^ and the destination point r*: **r**_0_^(*i*+1)^ = <**r**>^(*i*)^ − (<**r**>^(*i*)^ − r*)/(*N* − *i*), where *N* is the number of SMD steps, *i* is the SMD step index from 1 to *N*−1, and **r**_0_^(*N*)^ = r* is the final N^th^ step. This sequent positioning external potential *U*^ext^ pulls the atom towards its destined final position r^*^. For short displacements of atoms, such a choice of the external force possesses flexibility capable to avoid and overcome reasonable potential barriers appearing on the way to the final locations of the moved atoms. In all SMD transformations, *N* = 21, force constant *k* = 500 kcal/(mol Å^2^) for the restraining potential applied to the pulled atoms in the SMD transformations, and *k* = 80 kcal/(mol Å^2^) for the other restrained atoms (the retained protein core). The 10 ns MD simulation was performed for each SMD step to restore the local equilibrium after the shifts of the restraining potential of the moved atoms. The total SMD displacement of the moved atoms was from 2 to 3 Å; therefore, each shift consisted of about 0.1 to 0.15 Å per step. The total simulation time of SMD consisted of 10 × 21 = 210 ns (not including the preliminary equilibration run). The SMD implementation was tested by computing the potentials of the mean force acting between ions in water, such as the Na^+^–Cl^−^ interaction, which were reproduced in accordance with the previously published ones [86].

## 5. Conclusions

In the present study, the interactions of the transmembrane domain of cannabinoid receptor I with its agonist and antagonist ligands have been analyzed. It was concluded that there is a region of the stiff core of this domain where the agonist and antagonist interact with the receptor in a similar manner. Based on the known structures of the receptor in its active and inactive states, a superposition of these active and inactive structures within this region on each other highlighted the changes in the structural transformation of the receptor at the activation. The main indicator of the activation appears as a movement of transmembrane helix TM7 relative to this stiff core region which pushes transmembrane helix TM6 off, providing allosteric modulation for the G protein bound to CB_1_ inside the cell. A designed model of inactive apo CB_1_ sensitive to be activated in the MD simulations of agonist binding allowed us to analyze the mechanism of CB_1_—agonist interactions. This analysis confirms the role of these interactions in the shift of TM7 towards the stiff core. This mechanism of CB_1_ activation by the TM7 pressing has been verified via steered molecular dynamics of the TM7 displacement relative to the stiff core. It has led to the CB_1_ activation irrespective of the agonist bound, which suggests that this movement of TM7 can be regarded as a sufficient condition of the activation of this GPCR receptor.

## Figures and Tables

**Table 1:** List of ligands of CB_1_ chosen for docking.

Name	Structure	Type	Function
AM-11542^a^	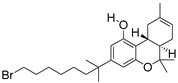	Phytocannabinoid analog	Agonist
A-834735^b^	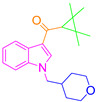	Alicyclic acylindole	Agonist
AM-2233	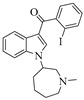	Benzoylindole	Agonist
JWH-203	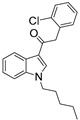	Phenylacetylindole	Agonist
Anandamide	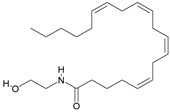	Endogenous	Agonist
∆9-THC	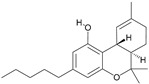	Native phytocannabinoid	Agonist
2-Arachidonoyl glycerol	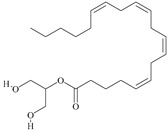	Endogenous	Agonist
CP-47497	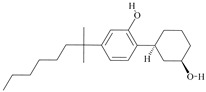	Cyclohexylphenol	Agonist
CP-47497-C8 homolog	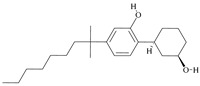	Cyclohexylphenol	Agonist
JWH-018	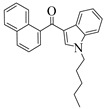	Naphtoylindole	Agonist
AM-251^c^	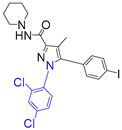	Diarylpirazole	Inverse Agonist
SR141716A	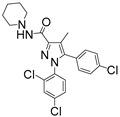	Diarylpirazole	Inverse Agonist
AM-281	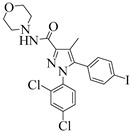	Diarylpirazole	Inverse Agonist
Taranabant	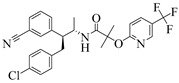	-	Inverse Agonist
Otenabant	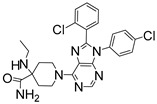	-	Inverse Agonist

^a^ ligand AM11542 from the crystal complex with CB_1_ [6]. ^b^ The ligand’s head is shown in green, the linker in orange, the core in magenta, and the tail in blue. ^c^ A subunit of the antagonist ligand that corresponds to agonists’ tails at binding is shown in blue.

**Table 2:** RMSD between simulated conformations of the receptor and its conformations in active and inactive states from crystal complexes.

Simulation Description	C_α_ RMSD/Max Distance (Å) from ^a^	
	Active	Inactive
5TGZ.pdb crystal complex with antagonist [7]	3.7/5.4	0.0/0.0
5XRA.pdb crystal complex with agonist [6]	0.0/0.0	3.7/5.4
5XR8.pdb crystal complex with agonist [6]	0.4/0.9	3.6/5.3
The model ^b^	4.1/5.7	0.9/1.6
The model (with no ligand) (400 ns) ^c^	4.1/5.4	1.3/2.0
The model with free AM11542 (400 ns)	1.3/2.2	2.9/4.3
The model with fixed ^d^ AM11542 (400 ns)	1.8/2.6	2.4/3.3
The model with fixed AM11542 without its head (200 ns)	3.1/5.2	1.7/2.5
The model with fixed AM11542 without its tail (200 ns)	2.7/3.6	2.2/3.1
The model with precisely fixed AM11542 without its tail (200 ns) ^e^	1.4/2.3	3.1/3.9
The model with free AM11542 without its tail (200 ns)	4.9/5.7	1.9/3.5
The model with free AM11542 with truncated C4 tail (200 ns)	4.4/5.9	1.7/2.9
The model with free AM11542 with truncated C5 tail (200 ns)	0.9/1.6	3.2/4.5
The model’s “reverse” simulations of the active complex obtained via 400 ns MD run of the model with AM11542: AM11542 is removed (400 ns)	3.5/4.9	2.1/3.4
5XRA.pdb crystal complex with AM11542 removed (200 ns)	0.7/1.3	3.8/5.5
The model with fixed AM11542, TM2 is fixed (200 ns)	3.2/4.7	1.5/2.6
The model’s “reverse” simulations of the active complex obtained via 400 ns MD run of the model with AM11542: AM11542 is removed along with Phe200 mutated to Gly200 (400 ns) ^f^	3.2/4.9	1.2/1.6
The model with fixed AM11542, Trp356 is mutated to Gly356 (200 ns)	2.2/3.9	2.0/3.0
The model with free AM11542, Trp356 is mutated to Gly356 (200 ns)	2.8/4.2	1.5/2.1
The model with free AM11542, Phe200 is mutated to Gly200 (200 ns)	3.6/4.8	1.4/3.0
The model with fixed AM11542, Ser383 is mutated to Gly383 (200 ns)	1.0/1.6	3.2/4.7
The model with free AM11542, Ser383 is mutated to Gly383 (200 ns)	3.1/4.5	1.4/2.3
The model with free AM11542, Leu359, and Met363 are mutated to Gly359 and Gly363 (200 ns)	1.6/2.4	3.0/4.4
The model with free AM11542, Phe379 is mutated to Gly379 (200 ns)	3.0/4.5	2.3/3.4

^a^ The comparison for residues 351–361 of TM6 and 381–391 of TM7 (Figure 8). ^b^ “The model” means the built model of inactive unliganded receptor, see text for further detail. ^c^ For all MD simulations, the structure averaged over the last 50 ns of trajectory is analyzed. ^d^ Fixation of ligand relative to fixed “basement”. ^e^ The location of the ligand’s head was taken from results of 400 ns MD simulations of the model with free AM11542. ^f^ Usually a mutation to alanine is applied for mutagenesis studies. For every experiment with a mutation to glycine, the integrity of the α-helix containing the mutated residue and stability of the α-helix backbone was verified.

**Table 3:** ∆∆G (kcal/mol) at binding AM11542 with truncated tail: ∆∆G = ∆G_2_ − ∆G_1_.

**Number of Tail Carbon Atoms**	0	3	4	5	8 (Br)
**2**					
**1**					
0					
3	6.22		−1.27		
4					
5		3.13	2.15		−5.40
8 (Br)	14.31	8.43 8.29			

**Table 4:** ∆G of residue transformation (kcal/mol). Transformations of one residue in the presence of other residue.

	**200**	Ala	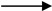	Phe
**356**				
Ala			−2.45	
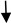		−6.94		−6.97
Trp			−3.12	

## Data Availability

Data sharing not applicable.

## Supplementary Materials

The following supporting information can be downloaded at: https://www.mdpi.com/article/10.3390/ijms241914874/s1.
