# Supplementary material for: Interaction of Synthetic Cannabinoid Receptor Agonists with Cannabinoid Receptor I: Insights into Activation Molecular Mechanism
Source: Int J Mol Sci. 2023 Oct 3;24(19):14874. doi: 10.3390/ijms241914874 (PMC10574015; doi:10.3390/ijms241914874)
Supplement: Supplementary file 1 [file ijms-24-14874-s001.zip › ijms-2614869-supplementary.pdf]

Interaction of Synthetic Cannabinoid Receptor Agonists with Cannabinoid Receptor I: Insights into Activation Molecular Mechanism

Sergei Gavryushov,[1,2*] Anton Bashilov,[2,3] Konstantin V. Cherashev-Tumanov,[2] Nikolay N. Kuzmich,[4] Tatyana I. Burykina,[2] and Boris N. Izotov[2]

[1] Engelhardt Institute of Molecular Biology, Russian Academy of Sciences, Vavilova str. 32, Moscow, 119991, Russia

[2] Sechenov First Moscow State Medical University, Institute for Translational Medicine and Biotechnology, 2-4 Bolshaya Pirogovskaya str., Moscow, 119991, Russia

[3] Skolkovo Institute of Science and Technology, Bolshoy Boulevard 30, Bld. 1, 121205, Moscow, Russia

[4] The Maurice and Vivienne Wohl Institute for Drug Discovery, Weizmann Institute of Science, Rehovot, 7610001, Israel

[*]corresponding author, email: sergei_gavryushov@yahoo.com

Supplementary Information

Table of Contents

S1. Metadynamics simulations

S2. Alternative way to build an apo-receptor model

S3. Alpha-carbon RMSD from simulations of the apo-receptor model of CB_1: demonstration of metastability of the model and the hydrophobic core chosen

S4. Superposition of the stiff cores for active and inactive conformations of 7TM domain of β_2-adrenergic receptor

S5. Superposition of the stiff cores for active and inactive conformations of 7TM domain of μ-opioid receptor

S6. Superposition of the stiff cores for active and inactive conformations of 7TM domain of rhodopsin

S1. Metadynamics simulations

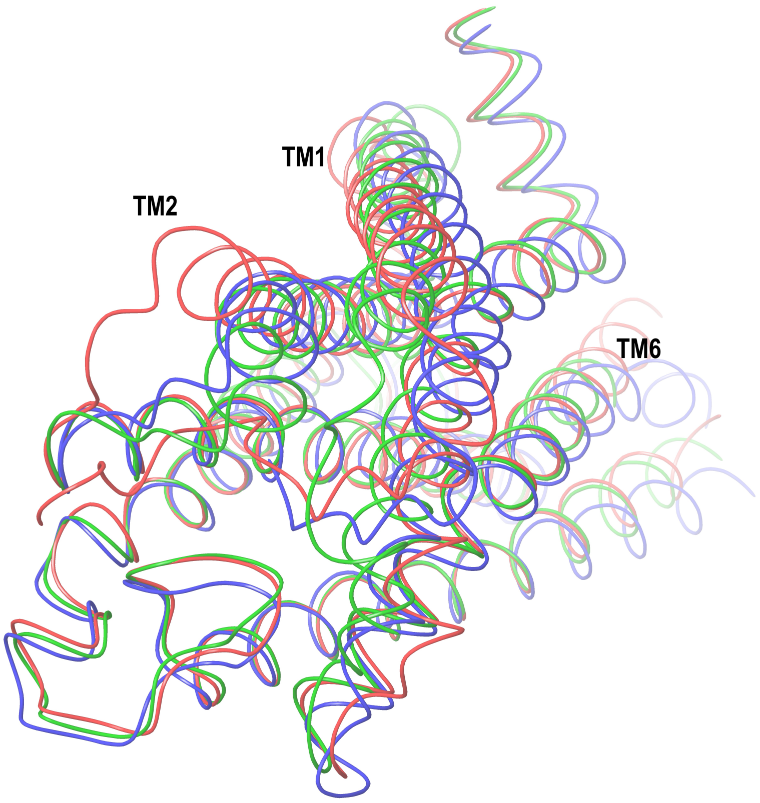

Figure S1. Superposition of the apo-receptor model of CB_1 described in the text (green), a similar model obtained from metadynamics simulations plus its 200ns MD run in the membrane environment (blue) and the structure of CB_1 from its crystal complex with antagonist ligand (red). The backbone chains of the structures are superimposed at the “basement” residues 195-199 (TM3), 243-249 (TM4), and 275-289 (TM5). According to placement of TM5, TM6, and TM7, all three structures correspond to the inactive state of the receptor.

S2. Alternative way to build an apo-receptor model

At first we built a chimeric model of the 7TM domain of CB_1, where the conformation of residues before Ala244 was taken from the active conformation (PDB ID: 5XRA) and residues after Ala244 were constructed according to receptor's inactive state (PDB ID: 5TGZ). It should be noted that between residues 243 and 249 both conformations can be superimposed at low RMSD of the backbone atoms (see above). In other words, TM1, TM2, TM3, and a part of TM4 were taken from the active state, whereas TM5, TM6, and TM7 from the inactive one. Such a structure can be easily built without serious interceptions of residue atoms if the backbone fitting involves the abovementioned fragments of TM3, TM4, and TM5 (marked in yellow in **Figure 5**).

The second step was equilibration of this chimeric model of apo receptor. It included a series of MD simulations of this model. Initial two simulation runs of 200 ns each were done at geometry restraints applied. At the first run, the "active-conformation" part of the backbone atoms was subjected to the restraining potential preventing their significant shift from initial positions. These simulations showed that the second part of the structure (after Ala244) had kept their inactive conformation. At the second 200 ns MD run, the "inactive-conformation" part was restrained in movement of the protein backbone atoms, whereas the protein chain preceding Ala244 was completely released. Its initial active conformation notably changed after the simulation. At the third 200 ns MD run, the entire 7TM domain of CB_1 was released. Anyway, after that α-helices TM5, TM6 and TM7 retained their inactive conformations such as observed in the crystal structure with antagonist ligand bound. The very shape of the protein has changed a little, except for α-helix TM1 appeared to be bent at one place. The final part of modeling included a slight correction of TM1 placement to restore its integrity. At the end the entire model was equilibrated again by means of a 200 ns MD run. And finally, the (meta)stability of this "apo-receptor model" was verified via a long-time MD run of 1000 ns length (all simulations in the membrane environment).

S3. Alpha-carbon RMSD from simulations of the apo-receptor model of CB_1: demonstration of metastability of the model and the stiff core chosen

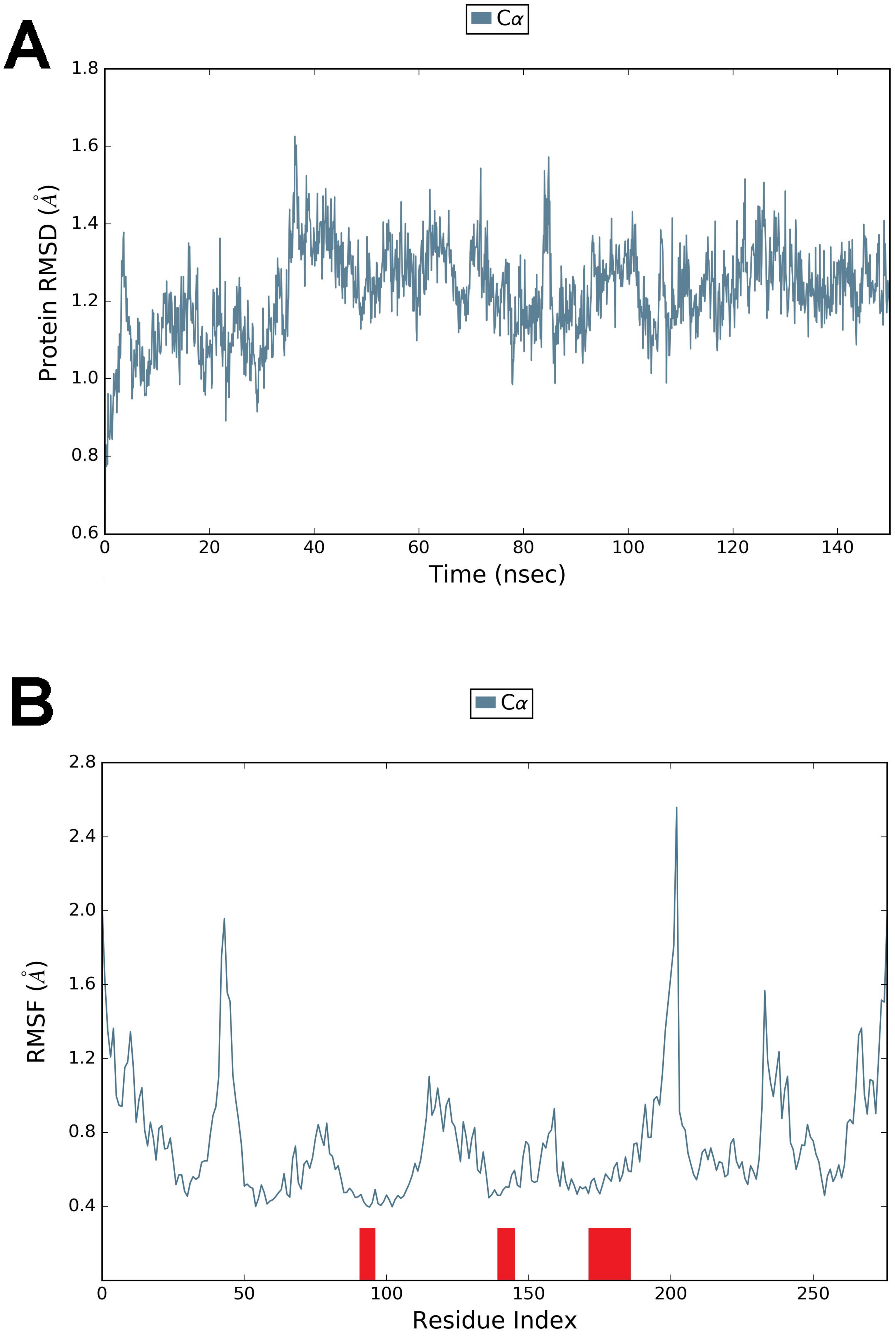

Figure S3. (A) RMSD averaged over all backbone carbon atoms for MD simulations of the apo-receptor model with agonist ligand bound during 450 – 600 ns of the simulation run. (B) RMSF for different alpha carbons of the backbone averaged in these simulations for the same simulation time: the fragments of the TM3, TM4 and TM5 (the "basement") chosen from the stiff core are marked in red.

S4. Superposition of the stiff cores for active and inactive conformations of 7TM domain of β_2-adrenergic receptor

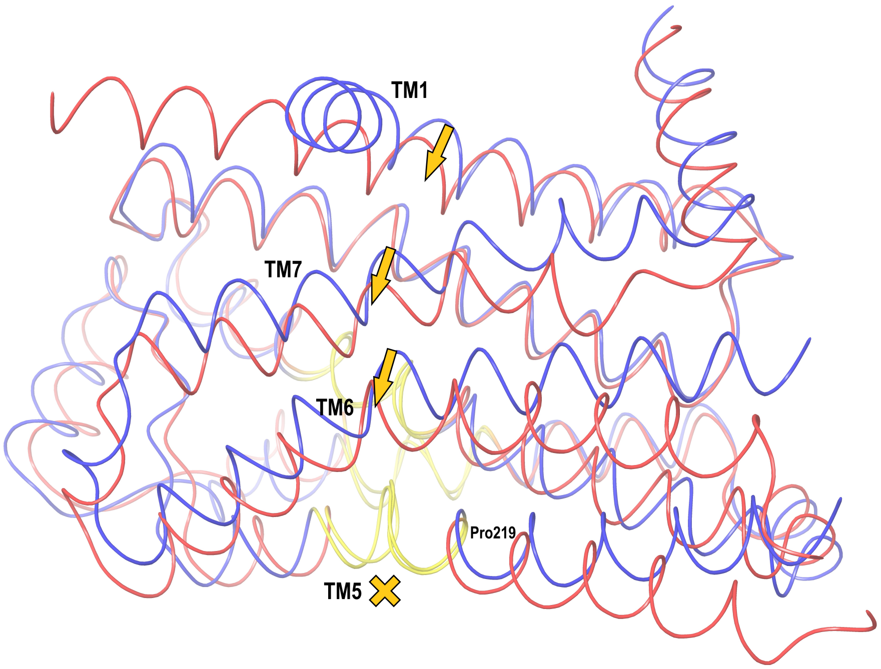

Figure S4. Superposition of thransmembrane domains of crystal complexes of β2-adrenergic receptor with its agonist ligand (PDB ID: 4LDE) and β1-adrenergic receptor with it antagonist (PDB ID: 2VT4). The first (active-state) structure is colored in red, the second (inactive-state) in blue. The backbone chains are superimposed to minimize RMSD for alpha carbons of TM3, TM4, and TM5 in the proximity to the Pro219 separating TM5 into its fixed and flexible parts (intervals of minimization are shown in yellow). The picture is quite similar to **Figure 8** of the text: movements of TM7 and TM6 in respect to the "basement" exceed 3 Å.

S5. Superposition of the stiff cores for active and inactive conformations of 7TM domain of μ-opioid receptor

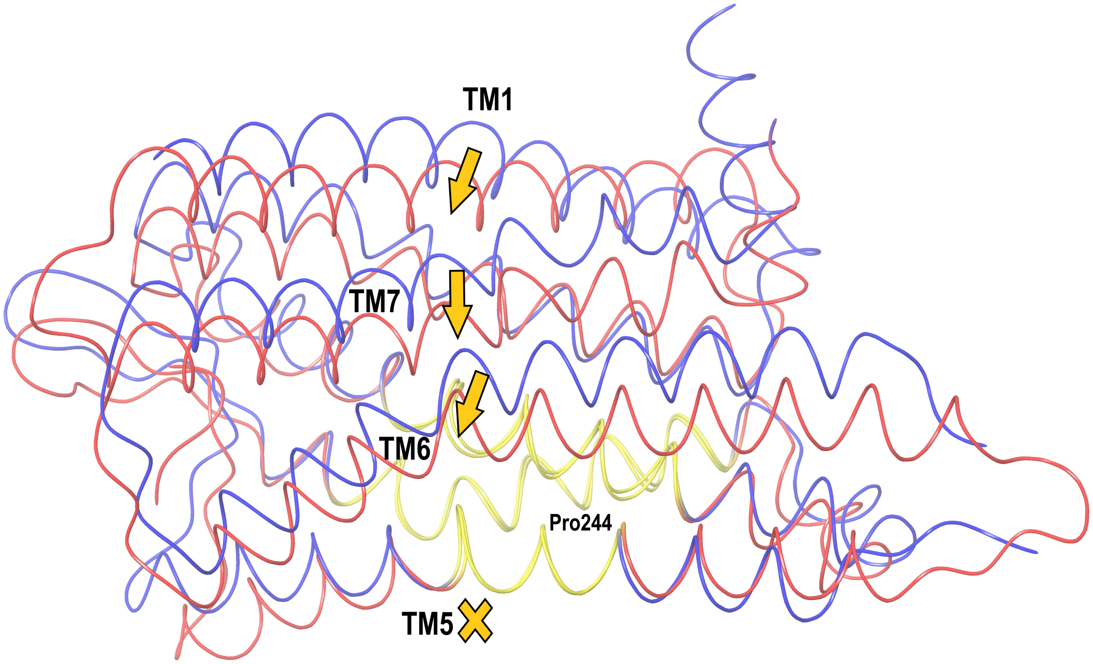

Figure S4. Superposition of thransmembrane domains of crystal complexes of μ-opioid receptor with its agonist ligand (PDB ID: 5C1M) and with it antagonist (PDB ID: 4DKL). The first (active-state) structure is colored in red, the second (inactive-state) in blue. The backbone chains are superimposed to minimize RMSD for alpha carbons of TM3, TM4, and TM5 in the proximity to the Pro244 separating TM5 into its fixed and flexible parts (intervals of minimization are shown in yellow). The picture is quite similar to **Figure 8** of the text: movements of TM7 and TM6 in respect to the “basement” exceed 3 Ǻ.

S6. Superposition of the stiff cores for active and inactive conformations of 7TM domain of rhodopsin

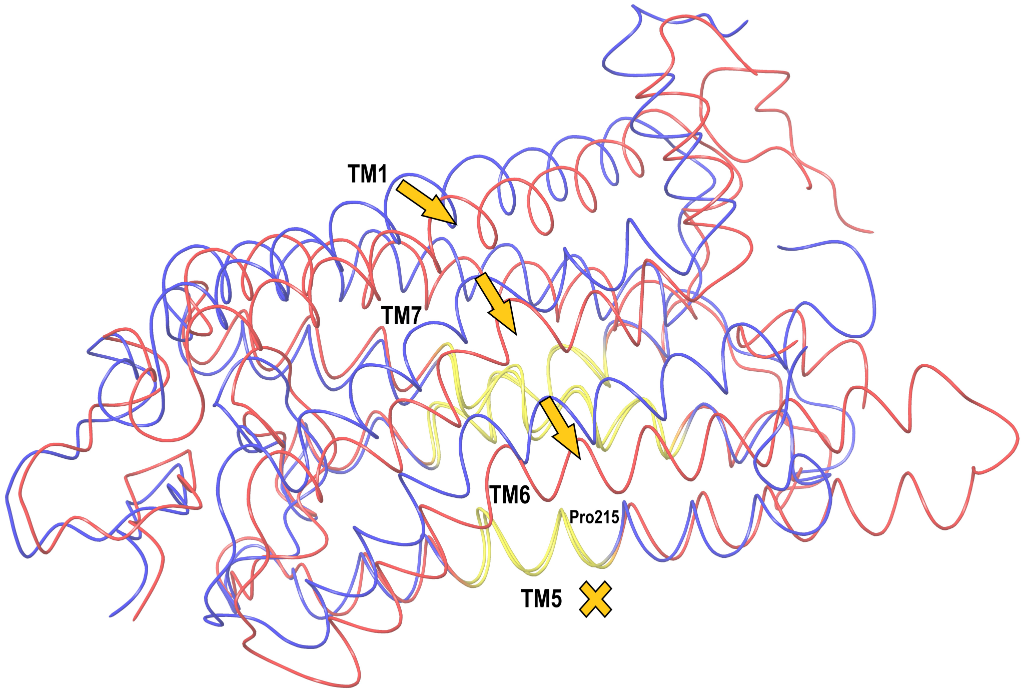

Figure S6. Superposition of thransmembrane domains of crystal structures of active metarhodopsin II (PDB ID: 3PQR) and inactive-state rhodopsin (PDB ID: 1F88). The first (active-state) structure is colored in red, the second (inactive-state) in blue. The backbone chains are superimposed to minimize RMSD for alpha carbons of TM3, TM4, and TM5 in the proximity to the Pro215 separating TM5 into its fixed and flexible parts (intervals of minimization are shown in yellow). The picture is quite similar to **Figure 8** of the text: movements of TM7 and TM6 in respect to the “basement” exceed 3 Ǻ.
